# Transcriptomic profiling reveals three molecular phenotypes of adenocarcinoma at the gastroesophageal junction

**DOI:** 10.1002/ijc.32384

**Published:** 2019-05-17

**Authors:** Jan Bornschein, Lorenz Wernisch, Maria Secrier, Ahmad Miremadi, Juliane Perner, Shona MacRae, Maria O'Donovan, Richard Newton, Suraj Menon, Lawrence Bower, Matthew D. Eldridge, Ginny Devonshire, Calvin Cheah, Richard Turkington, Richard H. Hardwick, Michael Selgrad, Marino Venerito, Peter Malfertheiner, on behalf of the OCCAMS Consortium, Ayesha Noorani, Rachael Fels Elliott, Paul A.W. Edwards, Nicola Grehan, Barbara Nutzinger, Jason Crawte, Hamza Chettouh, Gianmarco Contino, Xiaodun Li, Eleanor Gregson, Sebastian Zeki, Rachel de la Rue, Shalini Malhotra, Simon Tavaré, Andy G. Lynch, Mike L. Smith, Jim Davies, Charles Crichton, Nick Carroll, Peter Safranek, Andrew Hindmarsh, Vijayendran Sujendran, Stephen J. Hayes, Yeng Ang, Shaun R. Preston, Sarah Oakes, Izhar Bagwan, Vicki Save, Richard J.E. Skipworth, Ted R. Hupp, J. Robert O'Neill, Olga Tucker, Andrew Beggs, Philippe Taniere, Sonia Puig, Timothy J. Underwood, Fergus Noble, Jack Owsley, Hugh Barr, Neil Shepherd, Oliver Old, Jesper Lagergren, James Gossage, Andrew Davies, Fuju Chang, Janine Zylstra, Vicky Goh, Francesca D. Ciccarelli, Grant Sanders, Richard Berrisford, Catherine Harden, David Bunting, Mike Lewis, Ed Cheong, Bhaskar Kumar, Simon L. Parsons, Irshad Soomro, Philip Kaye, John Saunders, Laurence Lovat, Rehan Haidry, Victor Eneh, Laszlo Igali, Michael Scott, Shamila Sothi, Sari Suortamo, Suzy Lishman, George B. Hanna, Christopher J. Peters, Anna Grabowska, Rebecca C. Fitzgerald

**Affiliations:** ^1^ MRC Cancer Unit, Hutchison/MRC Research Centre University of Cambridge Cambridge United Kingdom; ^2^ Department of Gastroenterology, Hepatology and Infectious Diseases Otto‐von‐Guericke University Magdeburg Germany; ^3^ Translational Gastroenterology Unit Oxford University Hospitals Oxford United Kingdom; ^4^ MRC Biostatistics Unit University of Cambridge Cambridge United Kingdom; ^5^ Cancer Research UK Cambridge Institute University of Cambridge Cambridge United Kingdom; ^6^ Department of Histopathology, Addenbrooke's Hospital Cambridge University Hospitals Cambridge United Kingdom; ^7^ Department of Oncology Queen's University Northern Ireland United Kingdom; ^8^ Department of Surgery, Addenbrooke's Hospital Cambridge University Hospitals Cambridge United Kingdom

**Keywords:** gastric cancer, esophageal adenocarcinoma, gastroesophageal junction, gene expression profiling, Siewert classification

## Abstract

Cancers occurring at the gastroesophageal junction (GEJ) are classified as predominantly esophageal or gastric, which is often difficult to decipher. We hypothesized that the transcriptomic profile might reveal molecular subgroups which could help to define the tumor origin and behavior beyond anatomical location. The gene expression profiles of 107 treatment‐naïve, intestinal type, gastroesophageal adenocarcinomas were assessed by the Illumina‐HTv4.0 beadchip. Differential gene expression (limma), unsupervised subgroup assignment (mclust) and pathway analysis (gage) were undertaken in R statistical computing and results were related to demographic and clinical parameters. Unsupervised assignment of the gene expression profiles revealed three distinct molecular subgroups, which were not associated with anatomical location, tumor stage or grade (*p* > 0.05). Group 1 was enriched for pathways involved in cell turnover, Group 2 was enriched for metabolic processes and Group 3 for immune‐response pathways. Patients in group 1 showed the worst overall survival (*p* = 0.019). Key genes for the three subtypes were confirmed by immunohistochemistry. The newly defined intrinsic subtypes were analyzed in four independent datasets of gastric and esophageal adenocarcinomas with transcriptomic data available (RNAseq data: OCCAMS cohort, *n* = 158; gene expression arrays: Belfast, *n* = 63; Singapore, *n* = 191; Asian Cancer Research Group, *n* = 300). The subgroups were represented in the independent cohorts and pooled analysis confirmed the prognostic effect of the new subtypes. In conclusion, adenocarcinomas at the GEJ comprise three distinct molecular phenotypes which do not reflect anatomical location but rather inform our understanding of the key pathways expressed.

## Abstract 2

**What's new?**

Adenocarcinomas that arise at the junction between the esophagus and the stomach are currently classified based on location. Here, the authors looked at patterns of gene expression of these cancers. They found that gastro‐esophageal junction adenocarcinomas can be sorted into three biological subtypes, independent of location, based on gene expression. Group 1 cancers have boosted stomach‐specific genes that combat the effects of acid reflux. Group 2 tumors express genes characteristic to the intestinal tract, and the genes active in Group 3 relate to inflammation. The differences in biological pathway expression means that these differences could be used to improve treatment.

**Abbreviations**
ACRG: Asian Cancer Research Group
CIN: chromosomal instability group (subgroup of gastric cancers defined by the TCGA)
DDR: DNA‐damage repair
FDR: false discovery rate
GEJ: gastroesophageal junction
*H. pylori*: *Helicobacter pylori*
ICGC: International Cancer Genome Consortium
OCCAMS: Oesophageal Cancer Clinical and Molecular Stratification (study consortium)
TCGA: The Cancer Genome Atlas Consortium
WGS: Whole‐genome sequencing

## Introduction

Incidence of tumors at the gastroesophageal junction (GEJ) has increased rapidly over the past 50 years.^1^ Current clinical classification systems for these tumors are primarily based on the location of the main tumor mass and do not consider tumor biology.^2^ These systems have been developed to facilitate the decision making for the optimal surgical approach, which was historically the mainstay of treatment. With newly emerging systemic treatment options and multimodal therapy concepts being more dominant in curative treatment approaches, understanding of the biological processes that define different tumor subtypes is becoming increasingly important.

According to current knowledge, cancers in the distal part of the GEJ (Siewert Type 3) are more likely to arise from the proximal stomach.^3^, ^4^, ^5^, ^6^, ^7^ Proximal GEJ tumors (Siewert Type 1), on the other hand, are most likely of esophageal origin.^5^ It remains not clear if tumors originating directly from the GEJ (Siewert Type 2) comprise a mixed group of esophageal or gastric cancers or if these constitute a separate entity with distinct biological behavior. A recent study from *The Cancer Genome Atlas* (TCGA) consortium compared the genomic, epigenetic and transcript profiles of esophageal and gastric cancers comprising approximately 550 cancers.^8^ Interestingly, the authors concluded that esophageal, junctional and gastric adenocarcinomas are generally of a similar nature, with the majority of junctional cancers belonging to the chromosomal instability (CIN) subtype that has been described in their previous cohort of gastric cancers.^9^ CIN tumors were mainly intestinal‐type cancers according to the Laurén classification, as is expected for junctional cancers.^3^ Previous studies comparing junctional cancers to “true gastric” adenocarcinomas have often included diffuse‐type gastric tumors in the analyses introducing a bias due to the different cancer biology and a distinct genomic profile compared to intestinal type cancers.^9^ Previous molecular classifications were mainly based on the genomic features which do not necessarily reflect the active gene transcription landscape.

The primary aim of our study was to define adenocarcinomas at the GEJ according to their transcriptomic profile. Cases were very carefully selected to ensure that we had precise information on the location of the tumor in relation to the GEJ coupled with other clinical annotation. Since the Siewert classification is the current gold standard for clinical stratification of these tumors, we ensured that we had this information on each case in order to compare it with the molecular subtypes obtained.^10^ We also performed a pathway analysis of key expressed genes from each subgroup to further define the biological features of these subgroups and performed immunohistochemistry for selected genes to check expression at the protein level. The findings were confirmed in transcriptomic data from four independent datasets with clinical outcome data.

## Materials and Methods

### Study cohorts

All tissue samples were chemotherapy and radiotherapy‐naïve and prospectively collected either (*i*) as part of the Oesophageal Cancer Clinical and Molecular Stratification (OCCAMS) study consortium, coordinated by the University of Cambridge, United Kingdom, (*ii*) at the local tissue bank at Addenbrooke's Hospital, Cambridge University Hospitals (local ethics reference 10/H0305/1), or (*iii*) at the University of Magdeburg, Germany, Department of Gastroenterology, Hepatology & Infectious Diseases (local ethics references 132/01 and 34/08), before being retrospectively assessed for inclusion in our study. All patients gave written informed consent to tissue archiving and further analyses. The study was conducted in accordance with the Declaration of Helsinki. Tissue samples were obtained either during diagnostic endoscopy or surgical resection of the tumor. Diffuse‐type cancers and tumors with mixed pathology were excluded for the reasons explained in the Introduction.

A total of 84 patients with intestinal type adenocarcinoma at the GEJ as defined by Siewert and Stein in 1998 (35 GEJ1: main tumor mass 1–5 cm proximal to the junction, 31 GEJ2: 1 cm proximal to 2 cm distal to the junction, 18 GEJ3: 2–5 cm distal to the junction^10^) were included in two batches. For comparison, 23 nonjunctional gastric cancers (8 antrum, 15 gastric body) were included, as well as 11 mucosal biopsies from four noncancer controls (4 duodenum, 3 gastric body, 4 gastric cardia; local ethics reference LREC 01/149). Samples with histological evidence of squamous contamination as indicated by clear enrichment of genes associated with squamous differentiation were removed (*n* = 23) from the core analysis, leaving *n* = 61 GEJ cancers. Refer to Supporting Information Figure S1 for further details on the cohort selection process.

Four independent cohorts were used for validation purposes. The OCCAMS RNASeq cohort comprised 158 esophageal and GEJ adenocarcinomas. The “BELFAST” cohort included transcriptomic data from an additional 63 esophageal adenocarcinomas based on a modified Affymetrix expression array. The “SINGAPORE” cohort comprised 191,^11^ the “ACRG” (Asian Cancer Research Group) cohort of 300 true gastric cancers of Asian origin for comparison^12^ (see further details below).

### RNA and DNA extraction

Snap‐frozen tissue samples and matched blood, as a germline reference, were utilized. One section of the sample was stained with hematoxylin and eosin (H&E) and sent for cellularity review (≥70% tumor cellularity required for cancer samples) by at least two expert pathologists. Careful macrodissection and microdissection were performed to maintain this cellularity threshold.

RNA/DNA extraction was performed using the AllPrep kit (Qiagen, Hilden, Germany) and using the QIAamp DNA Blood Maxi kit (Qiagen, Hilden, Germany). RNA with an RNA integrity number (RIN) >7.0 was used for cDNA preparation (applying for material extracted from both biopsies and surgical resection specimens). Gene expression analysis was carried out on Illumina HT12 version 4.0 beadchip kit.

### Whole‐genome sequencing analysis

For 41 GEJ cases, whole‐genome sequencing (WGS) data was generated with 50× coverage for the cancer samples and 30× for germline reference samples as part of the International Cancer Genome Consortium (ICGC). Somatic mutations and indels were called using Strelka 1.0.13.^13^ Copy numbers were called using ASCAT‐NGS v2.1^14^ with the read counts at germline heterozygous positions as input for ASCAT being obtained using GATK 3.2‐2. Mutational signatures were identified using the methodology described by Alexandrov *et al*.^15^ To assess the alterations in DNA damage‐related pathways in our cohort, we performed an analysis similar to the one described by Pearl *et al*.^16^ Refer to the Supporting Information Methods for further details on the genomic analysis.

### RNA sequencing

For the OCCAMS validation cohort of 158 samples, transcriptome data was generated by RNA sequencing. Libraries were prepared using the Illumina TruSeq Stranded Total RNA Library Prep Kit and 75 bp paired‐end sequencing was performed using the HiSeq 4000 System. RNA‐seq data were aligned to the GRCh37_g1k reference genome using TopHat2. Aligned primary reads were then counted and normalized for gene length and sequencing depth. Log transformation of the expression data was performed as additional step of normalization before final analysis. Downstream analysis (see below) highlighted four outlier samples with extreme distribution of the gene expression pattern which were removed from further analysis resulting in 154 samples that were used for further validation.

### Immunohistochemistry

For a subset of 30 treatment‐naïve cancers for which surgical resection specimens were available (GEJ1: *n* = 4, GEJ2: *n* = 6, GEJ3: *n* = 6, body: *n* = 8, antrum: *n* = 6) immunohistochemical staining was performed on the Leica Bond‐II autostainer.

For all markers, a semiquantitative analysis of the cytoplasmatic staining was performed according to the modified immunoreactivity score by Remmele and Stegner multiplying the intensity of the cytoplasmatic staining (0: absent–3: strong signal) with the proportion of stained tumor cells (0: none–10: 100%).^17^

### Transcriptomic data analysis

All transcriptome data analyses were performed on R statistical computing using Bioconductor^18^ packages. All differential gene expression analyses were performed using *limma* in R.^19^ *p* Values for *limma*‐based differential gene expression analyses were adjusted for multiple comparison and represent false discovery rates (FDRs) for the respective tests. All unbiased group assignment was performed using *mclust* in R.^20^ Refer to Supporting Information Methods for further details.

The primary data sets for our study can be accessed as GSE96669. Publicly available datasets were used for validation: “BELFAST” dataset (E‐MTAB‐4666), the “SINGAPORE” cohort (GSE15459^11^) and the data of the Asian Cancer Research Group (“ACRG”; GSE66229^12^). Further datasets included were from colorectal (GSE38832), breast (GSE58812) and lung cancer samples (GSE31210).

## Results

### Comparison of the transcript profile of GEJ adenocarcinoma

**Figure 1:**
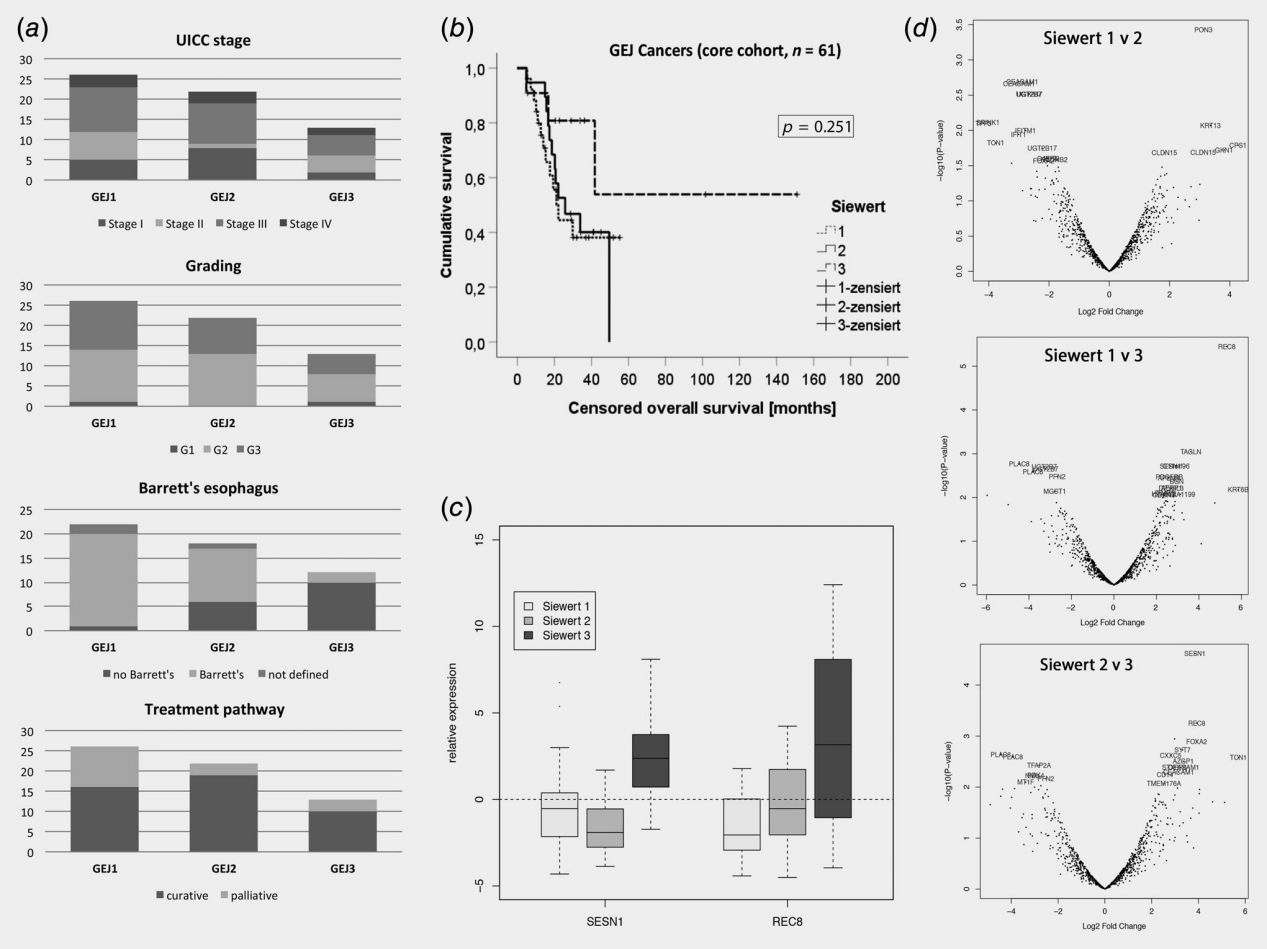
Comparison of clinical and gene expression data for GEJ cancers of different Siewert type. Panel (*a*) shows the distribution of UICC stage (*p* = 0.347), grading of the tumor (*p* = 0.823), presence of Barrett's esophagus (*p* < 0.001) and proportion of patients on a curative treatment pathway (*p* = 0.139) for GEJ Type 1, Type 2 and Type 3 cancers, respectively. There was no statistically significant difference in censored overall survival between cancers of different Siewert type as shown in (*b*). The boxplots in (*c*) show the relative expression of genes *REC8* and *SESN1*, which were the only differentially expressed genes in pairwise differential gene expression comparison of GEJ cancers. Panel (*d*) shows the respective volcano plots for the differential gene expression analyses.

Sixty‐one junctional adenocarcinomas across all three Siewert types (GEJ1: 26, GEJ2: 22, GEJ3: 13) were included in the core analysis. There was no significant difference in clinical parameters between the Siewert types, apart from an expected higher proportion of Barrett's esophagus in patients with GEJ1 cancers. Patients underwent standard clinical treatment pathways according to their stage (Fig. 1 *a*) and there was no significant difference in median survival between GEJ1 (22.2 m), GEJ2 (25.9 m) and GEJ3 (29.9 m) tumors (*p* = 0.251; Fig. 1 *b*).

**Figure 2:**
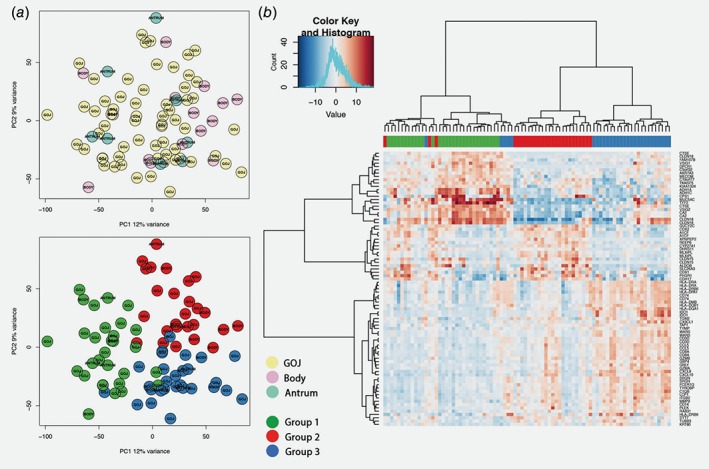
Gene expression profile of different subgroups of junctional and nonjunctional intestinal‐type adenocarcinomas. Panel (*a*) shows the principal component plots for the distribution of the samples according to the first two principal components of the gene expression analysis. The top panel shows the distribution according to location of the main tumor mass, the bottom panel the subgroups as identified by *mclust* (the color code is displayed in the bottom middle). The heatmap in panel (*b*) illustrates the clustering of the new subtypes (group 1: green, group 2: red, group 3: blue). Displayed are the combined group of 61 GEJ and 23 nonjunctional cancers (columns) and the target set of 67 genes (rows, see main text for details).

Differential gene expression analysis between tumors of different Siewert types using *limma* 19 revealed that *REC8* (REC8 Meiotic Recombination Protein) was the only gene with differential expression when comparing between GEJ1 and GEJ3 tumors (FDR: *p* = 0.004), and *SESN1* (Sestrin‐1) between GEJ2 and GEJ3 tumors (FDR: *p* = 0.024). There were no differentially expressed genes below the threshold of *p* = 0.01 when GEJ1 tumors were compared to GEJ2 cancers (Figs. 1 *c* and 1*d*; Supporting Information Table S1). Furthermore, there were no differentially expressed genes between junctional and nonjunctional cancers (Supporting Information Table S1). When the first two principal components of the transcript profile of these 84 gastroesophageal cancers were displayed, a random distribution was observed according to the anatomical location (Fig. 2 *a*).

Next, we applied an unbiased approach to identify molecularly intrinsic cancer subtypes. Using the *mclust* algorithm,^20^ an optimal solution of three distinct subgroups for the core cohort of 61 GEJ cancers emerged (Supporting Information Fig. 2 *a*). Patients were thus assigned by *mclust* to three subgroups and a group‐by‐group differential gene expression analysis was performed to identify genes defining each subtype (Supporting Information Table S2). Of these, 82 genes with a *p*‐score <0.0001 (Supporting Information Methods) were considered as candidates for discrimination between the new subtypes. Since location had no impact on the analysis of differentially expressed genes between tumors of different Siewert types and junctional *vs*. nonjunctional cancers, we also performed a combined analysis with the 23 nonjunctional gastric tumors which resulted in a similar three group distribution (Fig. 2 *a*, Supporting Information Fig. S2 *b*). Of the genes mentioned above, 67 genes (82%) were also represented in this parallel analysis which were then selected for further validation (Fig. 2 *b*, Supporting Information Methods).

Since GEJ cancers can express a range of intestinal cell types, we also compared the gene expression profile of the identified subtypes with samples from gastric and duodenal mucosa of patients without cancer. Compared to these noncancer mucosal controls, upregulation of cancer‐specific genes was confirmed but no further genes were highlighted (Supporting Information Table S3).

**Figure 3:**
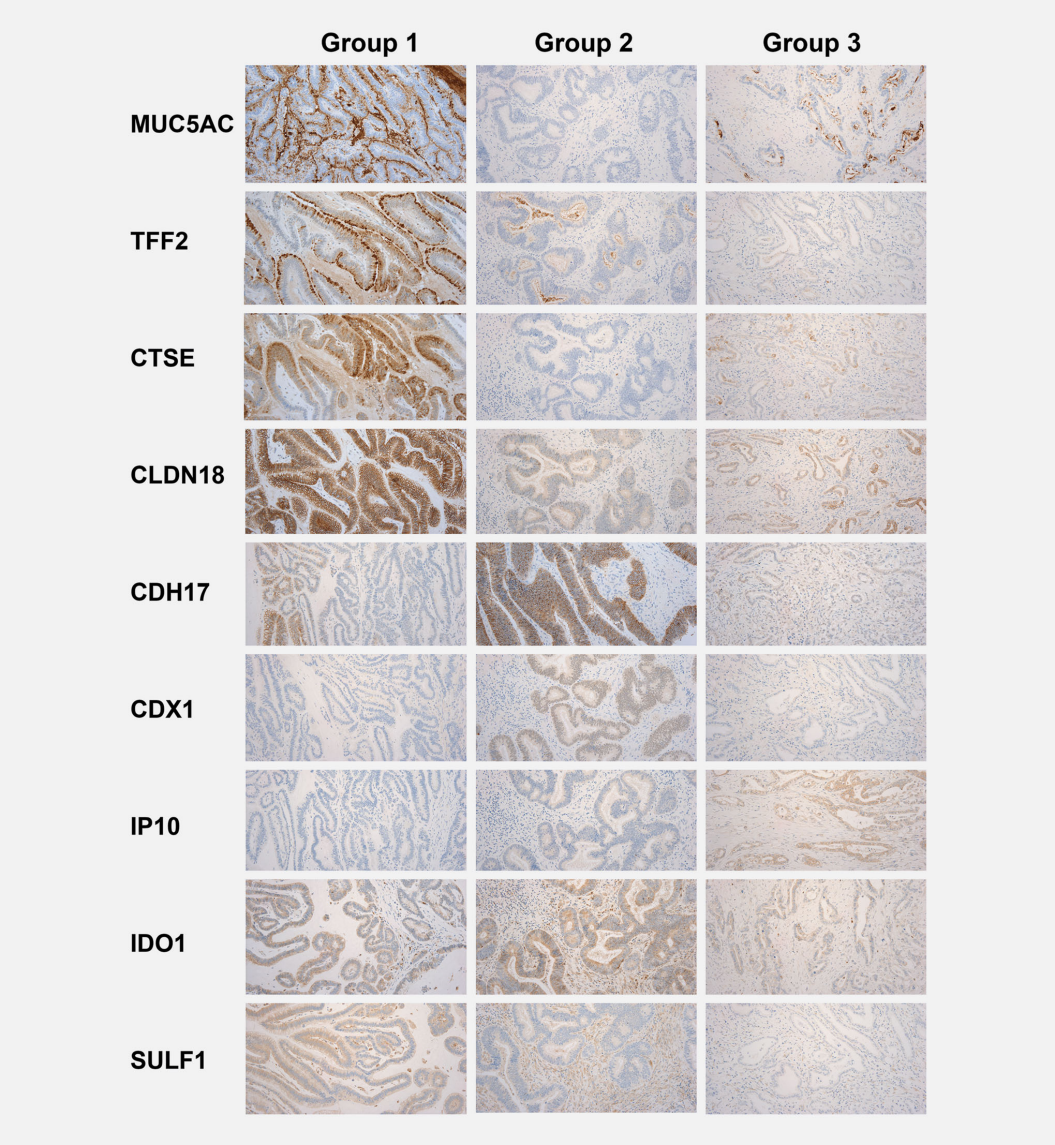
Immunohistochemistry profile of the three subtypes of gastroesophageal adenocarcinoma. The immunohistochemical staining for markers that were ranked highest in the principal component analysis is shown for the respective groups. One representative case for each group is displayed. For some of the markers, distinction was more obvious (e.g., CTSE more strongly expressed in Group 1, and CDH17 more strongly expressed in the Group 2), whereas for some markers differences were subtler (e.g., nuclear staining of CDX1 in Group 2 or cytoplasmic staining of IP10 in Group 3). For MUC5AC cytoplasmic staining and extracellular mucin is assessed, for CTSE, and IP10 cytoplasmic staining is typical, for CLDN18 and CDH17 membranous staining, and for CDX1 nuclear staining.

Thirty patients for which high‐quality surgical resection specimens were available were selected for immunohistochemistry to investigate if the new subtypes could also be confirmed at the protein level. Markers were selected according to the first and second principal component of the gene expression data analysis (Fig. 3).

The immunostaining scores for all markers were as expected for each subgroup (Supporting Information Table S4). For CTSE (*p* = 0.047) and membranous CLDN18 (*p* = 0.048), the absolute scores were significantly different between the three subgroups with the highest scores for Group 1. SULF1 (a marker for stromal activation) was more intensely stained in patients of Group 1 and Group 2 (*p* = 0.004). Presence of IDO1 positive immune cells was highest in Group 3 tumors (90%; *p* = 0.217) and was associated with IP10 expression in the tumor (*p* = 0.017).

### Pathway analysis support different biological background of the three subtypes

In order to better understand the biological pathways underpinning the new group assignment, gene‐set enrichment analysis was performed using *gage* in R.^21^ Based on KEGG terms, the top essential pathways enriched in Group 1 were “Ribosome,” “Fatty Acid Metabolism,” “Oxidative Phosphorylation” and pathways involved in nucleic acid turnover (both DNA and RNA). Group 2 was characterized by “Steroid Hormone Biosynthesis,” “Peroxisome,” “Primary Bile Acid Biosynthesis” and terms related to metabolic processes. Essential KEGG pathways enriched in Group 3 were “Antigen Processing and Presentation,” “Chemokine Signaling Pathways” and “Natural Killer Cell‐Mediated Cytotoxicity,” among other immune‐response related terms (Table 1; Supporting Information Table S4). These results were in line with a parallel analysis based on gene ontology terms (Supporting Information Table S5).

**Table 1:** Gene‐set based pathway analysis based on *Kegg* terms for the three intrinsic subgroups

Group 1			Group 2			Group 3		
*Kegg pathway*	*p‐value*	*q‐value*	*Kegg pathway*	*p‐value*	*q‐value*	*Kegg pathway*	*p‐value*	*q‐value*
**Ribosome**	<0.001	<0.001	**Steroid hormone biosynthesis**	<0.001	<0.001	**Antigen processing and presentation**	<0.001	<0.001
**Fatty acid metabolism**	<0.001	<0.001	**Peroxisome**	<0.001	<0.001	Phagosome	<0.001	<0.001
**Oxidative phosphorylation**	<0.001	<0.001	**Primary bile acid biosynthesis**	<0.001	0.001	**Chemokine signaling pathway**	<0.001	<0.001
**Metabolism of xenobiotics by cytochrome P450**	<0.001	<0.001	**Fat digestion and absorption**	0.001	0.025	Cell adhesion molecules (CAMs)	<0.001	<0.001
Retinol metabolism	<0.001	<0.001	**Drug metabolism—other enzymes**	0.001	0.035	**Natural killer cell mediated cytotoxicity**	<0.001	<0.001
**Valine, leucine and isoleucine degradation**	<0.001	<0.001	**Carbohydrate digestion and absorption**	0.001	0.035	Intestinal immune network for IgA production	<0.001	<0.001
Drug metabolism—cytochrome P450	<0.001	<0.001	**Renin‐angiotensin system**	0.002	0.040	**Osteoclast differentiation**	<0.001	<0.001
Propanoate metabolism	<0.001	0.001	**Vitamin digestion and absorption**	0.002	0.040	**Toll‐like receptor signaling pathway**	<0.001	<0.001
**Glycolysis/Gluconeogenesis**	<0.001	0.001	**Citrate cycle (TCA cycle)**	0.003	0.051	**Haematopoietic cell lineage**	<0.001	<0.001
**RNA transport**	<0.001	0.001	**Starch and sucrose metabolism**	0.005	0.083	**ECM‐receptor interaction**	<0.001	<0.001
**Nitrogen metabolism**	<0.001	0.001	**Glutathione metabolism**	0.007	0.090	Focal adhesion	<0.001	<0.001
**Glyoxylate and dicarboxylate metabolism**	<0.001	0.004	**Steroid biosynthesis**	0.007	0.090	**NOD‐like receptor signaling pathway**	<0.001	<0.001
**DNA replication**	<0.001	0.004	**Sulphur relay system**	0.007	0.090	**Leukocyte transendothelial migration**	<0.001	<0.001
**Pentose and glucuronate interconversions**	<0.001	0.005	PPAR signaling pathway	0.011	0.130	**T‐cell receptor signaling pathway**	<0.001	<0.001
Butanoate metabolism	0.001	0.006	Glycerolipid metabolism	0.013	0.144	**Complement and coagulation cascades**	<0.001	<0.001
**Base excision repair**	0.001	0.006	Other types of O‐glycan biosynthesis	0.024	0.220	Regulation of actin cytoskeleton	<0.001	<0.001
Mismatch repair	0.001	0.008	Dorso‐ventral axis formation	0.024	0.220	**Jak–STAT signaling pathway**	<0.001	<0.001
**Tyrosine metabolism**	0.001	0.009	Fructose and mannose metabolism	0.024	0.220	**MAPK signaling pathway**	<0.001	<0.001
Pyruvate metabolism	0.002	0.015	Ascorbate and aldarate metabolism	0.027	0.227	**Lysosome**	<0.001	<0.001
**RNA degradation**	0.003	0.022	Metabolism of xenobiotics by cytochrome P450	0.029	0.238	**RIG‐I‐like receptor signaling pathway**	<0.001	<0.001

Essential core‐pathways are printed in bold. Analysis was done using *gage* in R.

A complementary *Ingenuity® Pathway Analysis* (IPA®, QIAGEN Redwood City, http://www.qiagen.com/ingenuity) showed broadly similar results (Supporting Information Table S6). The expression profile of Group 1 was associated with canonical pathways involved in the degradation of organic substances, with the top regulatory networks being related to fatty acid metabolism. Group 2 showed enrichment for genes involved in retinoic acid receptor activation, bile acid biosynthesis and endothelin signaling. Group 3 was characterized by canonical pathways involved in immune response and cell–cell interaction.

### Association of the three subtypes to clinical and genomic parameters

**Figure 4:**
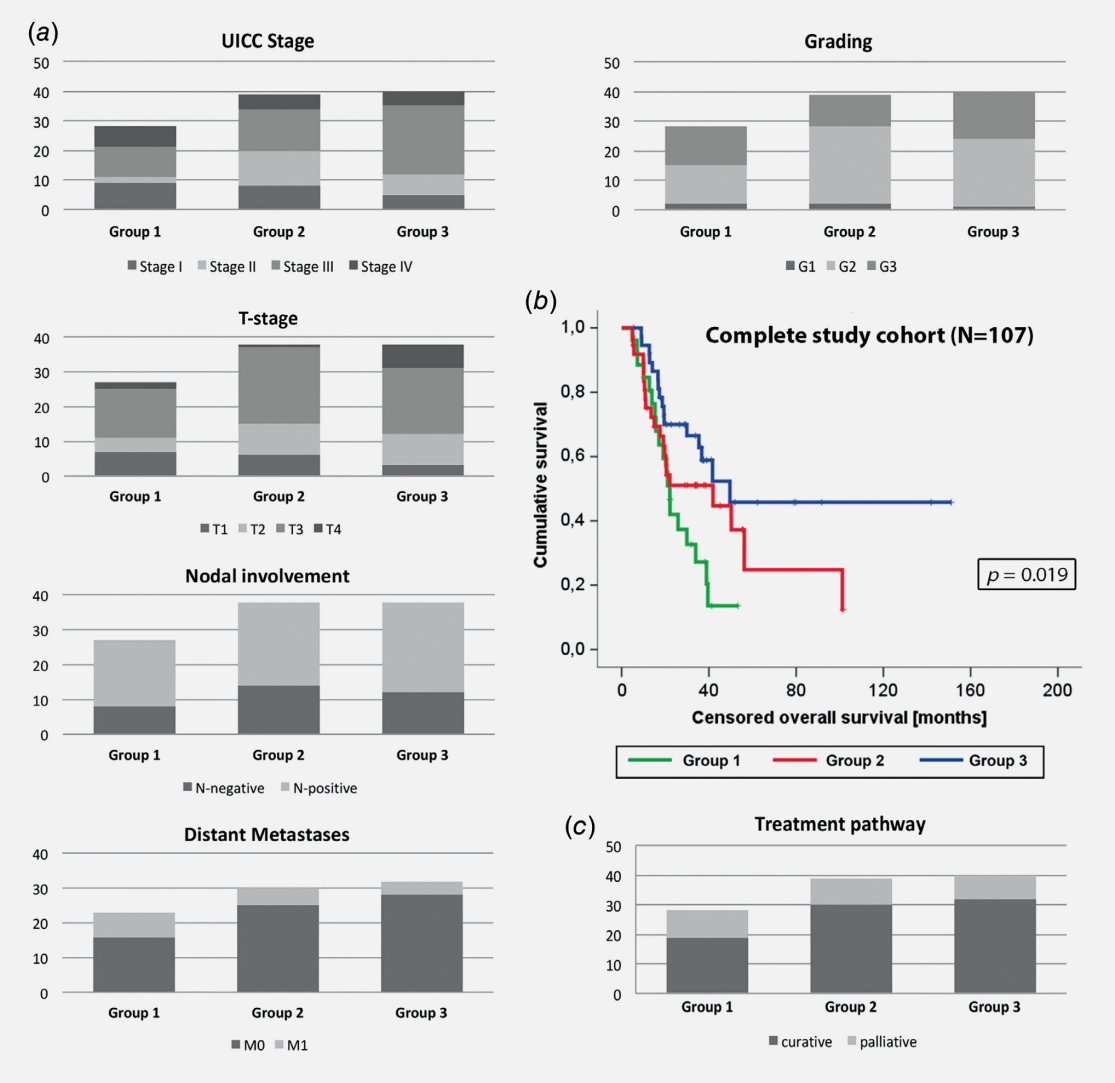
Comparison of clinicopathological data and overall survival for the new subgroups based on the whole study cohort (*n* = 107). Panel (*a*) shows the distribution of UICC stage (*p* = 0.058), T‐stage (*p* = 0.178), nodal involvement (*p* = 0.865), presence of distant metastases (*p* = 0.234), as well as grading of the tumor (*p* = 0.451) for the new subgroups. Censored overall survival for each subgroup is shown in the Kaplan–Meier graph in (*b*) with group one showing the worst and Group 3 the best prognostic outcome. The proportion of patients on a curative treatment pathway for each group (*p* = 0.531) is displayed in (*c*).

Next, we assessed whether there was any association between the new subtypes and clinical parameters. Of 107 cancers, 28 (26.2%) were assigned to Group 1, 39 (36.4%) to Group 2 and 40 (37.4%) to Group 3. Overall, there was no relevant difference between the groups with regards to clinical or demographic factors (Fig. 4 *a*; Supporting Information Table S7). When only patients with cancer at the GEJ were analyzed, there was a strong association of the presence of Barrett's esophagus with the new subgroups (Group 1: 93.3%, Group 2: 60.7%, Group 3: 40.9%; *p* = 0.004).

Kaplan–Meier analysis revealed a difference in the median overall survival between the three subtypes, with borderline statistical significance (Group 1: 25.9 m *vs*. Group 2: 45.2 m *vs*. Group 3: 83.5 m; *p* = 0.019; Fig. 4 *b*) compared to the other known clinical parameters: stage of disease (*p* < 0.001), T‐stage (*p* < 0.001), nodal involvement (*p* < 0.001) and presence of distant metastases (*p* < 0.001).

Cox regression analysis showed that the new tumor subtype was an independent prognostic factor for overall survival with a Hazard ratio of 1.506 (95% confidence interval: 1.021–2.222; *p* = 0.039), along with nodal involvement and distant metastases. There was no difference in the proportion of patients who underwent a curative or a palliative treatment pathway between each group (Fig. 4 *c*, Supporting Information Table S7).

For 41 cases, WGS data were available to compare the genomic properties of the new subtypes. Bearing in mind the heterogeneous nature of genomic alterations in this cancer and the relatively small sample size with WGS available,^22^ there was no demonstrable difference between the three subtypes with regards to the overall mutational burden and the profile of copy number aberrations and amplifications or deletions (Supporting Information Fig. 3 *a*). There was enrichment across all groups for mutational signatures 1, 2, 3 and 17 as defined by Alexandrov *et al*.^15^ (Supporting Information Fig. 3 *b*), which was as expected for gastroesophageal adenocarcinomas.^22^ Group 3 showed a slightly higher prevalence for alterations in genes involved in DNA damage repair (DDR) pathways (checkpoint factors, chromatin remodeling, Fanconi anemia, telomere maintenance, translesion synthesis; Supporting Information Fig. 3 *c*). In keeping with this, this subgroup also showed a higher proportion of “DDR impaired” positive tumors according to the classification recently published by our group^22^ although it did not reach statistical significance due to the relatively small numbers with WGS data available (Supporting Information Figs. 4 *a* and 4 *b*).

### Application of new subtype classification in independent cohorts

It is crucial to determine if these findings are reproducible in other datasets across other platforms. Four further datasets were available for analysis. These were not necessarily focused on junctional tumors but demonstrate the broad applicability of these molecular subgroups to esophageal and gastric adenocarcinomas independent of their anatomical location. While the OCCAMS dataset was generated based on RNA‐sequencing, the BELFAST, SINGAPORE and ACRG datasets were generated on Affymetrix platforms. The 67 genes panel was applied to all four validation cohorts (Supporting Information Methods) for subtype assignment.

**Figure 5:**
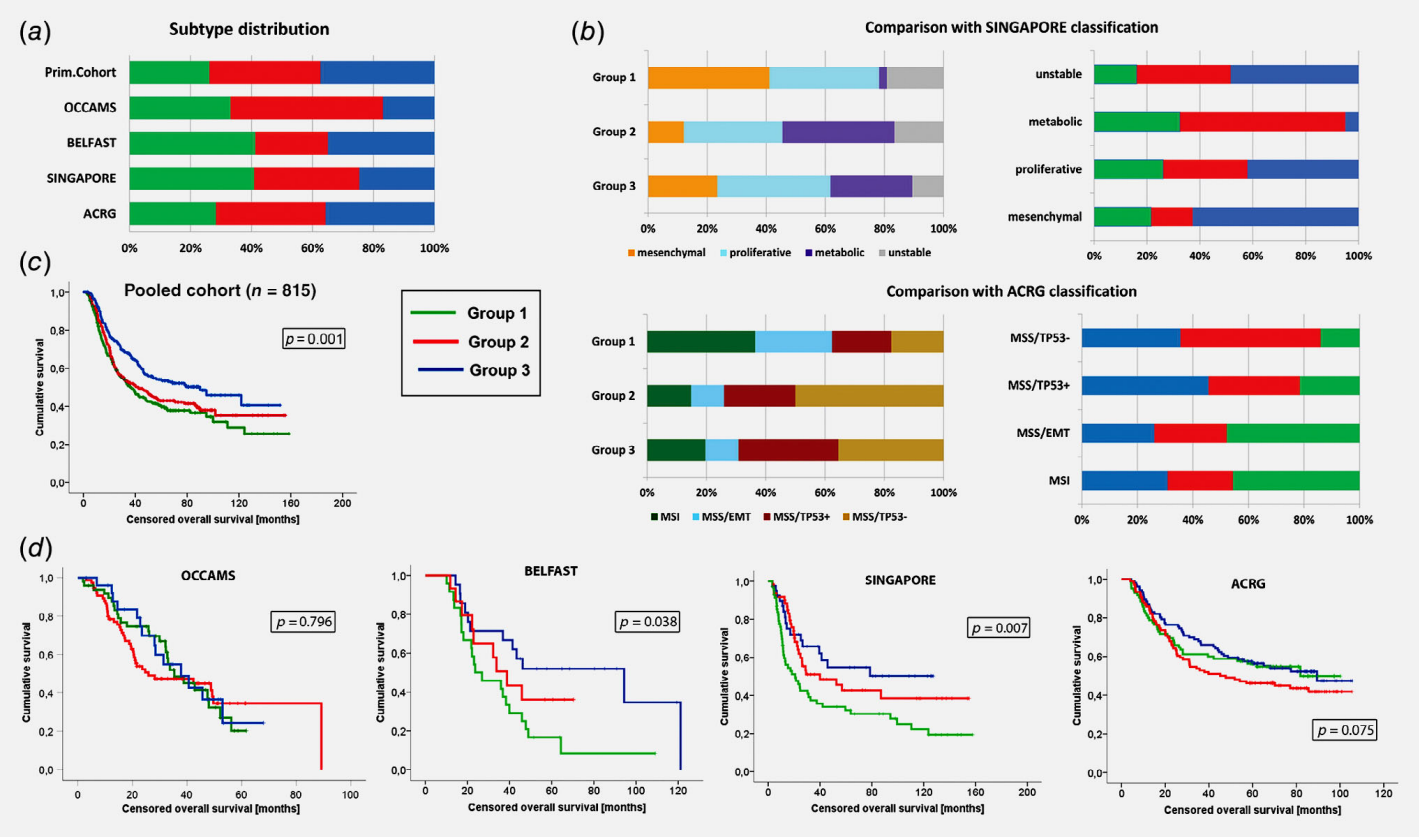
Comparison of subtype distribution and survival in independent cohorts. Panel (*a*) shows the distribution of each subtype in our primary cohort and across the four validation cohorts (please see main text for further details). We also compared the group stratification as originally published for the SINGAPORE and ACRG cohorts (*b*). On the left we show the distribution of the originally published subtypes within our new groups for each cohort, on the right the distribution of our newly defined subtypes within each subtype that has previously been published by Lei *et al*. and (top) Cristescu *et al*. (bottom). Despite a significant statistical overlap between the different group stratifications, there are still considerable differences in the distribution. The Kaplan–Meier curve in (*c*) shows the cumulative overall survival (in months) for each of the new subtypes in the pooled cohort of all 815 patients across all five subcohorts (Group 1: green, Group 2: red, Group 3: blue). The Kaplan–Meier curves below (*d*) show the outcome for each of the validation cohorts.

The 154 samples of the OCCAMS cohort recapitulated a three‐group solution as expected (Group 1: *n* = 51, Group 2: *n* = 77, Group 3: *n* = 26), which was also the case for the 63 esophageal adenocarcinomas of the BELFAST cohort (Group 1: *n* = 26, Group 2: *n* = 15, Group 3: *n* = 22). The 191 gastric adenocarcinomas from the SINGAPORE cohort^11^ (tumors of unclear histological subtype were excluded), could also be classified into the three groups (Group 1: *n* = 78, Group 2: *n* = 66, Group 3: *n* = 47); and subtype assignment was also consistent for the 300 gastric cancers of the Asian Cancer Research Group (ACRG)^12^ (Group 1: *n* = 85, Group 2: *n* = 108, Group 3: *n* = 107; Fig. 5 *a*). For the latter two Asian validation cohorts, our classification showed statistical overlap (*p* < 0.001) with the subtypes that have been previously proposed by Lei *et al*.^11^ and Cristescu *et al*.,^12^ but the distribution of the subtypes within the cohorts suggested a distinct classification (Fig. 5 *b*). Ethnic origin did not influence the results since there was no difference in the subtype distribution between Western (OCCAMS, BELFAST and primary study cohort) and Asian (SINGAPORE, ACRG) patients (*p* = 0.967). This was also the case when cohorts with predominantly esophageal cancers were compared to gastric tumor cohorts (*p* = 0.351).

A pooled analysis of all 815 cases across all five cohorts (including our primary study cohort) showed significantly different median overall survival, with Group 1 showing the worst and Group 3 the best prognosis (*p* = 0.001). Similarly to our primary cohort, also grade of differentiation (*p* < 0.001), UICC stage (*p* < 0.001), nodal involvement (*p* < 0.001) and distant metastases were influencing factors (*p* < 0.001). Cox regression analysis including stage, grading and the new subtypes as factors confirmed both stage of disease (*p* < 0.001) and the new subtypes (*p* = 0.002) as independent prognostic factor, whereas grading was not confirmed (*p* = 0.169). In the individual validation cohorts, a moderate statistical difference in outcome could be seen in the BELFAST (*p* = 0.038) and the SINGAPORE (*p* = 0.007) cohort, but not in the ACRG (*p* = 0.075) and OCCAMS (*p* = 0.796) datasets (Fig. 5 *d*).

To check whether the findings were cancer type specific we applied our gene panel to datasets from other tumor entities (colorectal, lung and breast) which interestingly also clustered into three groups suggesting that there may be some modules common across multiple cancer types, but they did not show differences in survival (Supporting Information Fig. 5).

## Discussion

These data confirm that the biological properties of adenocarcinomas at the GEJ are independent of the anatomical location of the main tumor mass. Adenocarcinomas at the GEJ and nonjunctional gastric cancers of the intestinal type can be stratified into three biologically distinct subtypes based on their gene expression profile.

The pathway analysis gives some insight into the biological basis for each tumor subtype. Group 1 shows features which appear to be in keeping with mucosal damage by reflux components including enrichment of stomach‐specific genes, particularly *CLDN18* which is upregulated under reflux conditions to increase mucosal resistance to acid^23^ and *MUC5AC* which is upregulated in response to bile exposure.^24^ In addition, the metabolic processes enhanced in this group indicate a possible interaction with visceral adipocytes. Adipose tissue can constitute a proinflammatory microenvironment in obese patients, leading to stromal activation which is associated with more aggressive tumor behavior and poor prognosis.^25^, ^26^, ^27^, ^28^, ^29^ Negative regulators of adipogenesis like BMP and activin membrane‐bound inhibitor (BAMBI) or transglutaminase 2 (TGM2) showed the lowest expression in Group 1 (Supporting Information Fig. S6).^30^, ^31^

Group 2 is characterized by metabolic pathways which are usually active in the intestinal and hepatobiliary tract. Expression of the intestinal transcription factor *CDX2* can also be induced by exposure to bile acids, mediated by the farnesoid X receptor.^32^ The intestinal properties of Group 2 are further supported by expression of Achaete‐scute family bHLH transcription factor 2 (ASCL2), an intestinal stemness marker (Supporting Information Fig. S6).

Group 3 is linked to inflammatory response regulation showing a threefold to fivefold higher expression of *CD8A* (T‐cell marker CD8) and *GZMB* (granzyme B, marker of cytotoxic activity) compared to the other groups (Supporting Information Fig. S6). Gastric cancers with a high ratio of tumor‐infiltrating lymphocytes show a better prognosis and are associated with impairment in mismatch repair pathways.^33^ DDR impairment can also be associated with chronic infection with *H. pylori*,^34^ and is a feature of a subtype of esophageal adenocarcinomas with a higher mutational and neo‐antigen burden.^22^ While the small subcohort for which WGS data were analyzed showed a trend toward a higher proportion of “DDR impaired” tumors^22^ in Group 3, this association was not confirmed in the OCCAMS validation cohort. In this cohort, Group 2 tumors showed a higher proportion of the “DDR impaired” genome signature type. It is of note that there is some overlap between the dominant genes for Group 2 and Group 3 (Fig. 2). It requires further elucidation in larger cohorts to determine whether our transcriptome‐based classification is linked to genome‐based subtypes.

We also assessed the association of our new subgroups to MSI status using data from the OCCAMS cohort for which WGS data was available. MSI status was classified as MSI stable (MSS) or MSI‐low/high (MSI‐L/H) as described before.^22^ While 91.4% of patients were classified as MSS, 8.6% were MSI‐L/H and there was no association of MSI status to the new subgroups (*p* = 0.361). The low prevalence of MSI positive cases is in keeping with previous reports for this disease.^9^ However, we also compared MLH1 status that was provided for the ACRG cohort with the new subtype classification. Of 300 cases 23.1% were MLH1‐negative indicating MSI‐H status. This was more often seen in Group 3 (32.9%) when compared to groups 1 (18.7%) and Group 2 (14.8%; *p =* 0.007). Although there is some overlap, MSI status affects only about a third of patients in Group 3 and is therefore unlikely to be a dominant discriminating factor for our classification.

Interestingly, there is a strong association between the new subgroups and presence of Barrett's esophagus. If only patients with junctional cancers were analyzed, there was a dominance of Barrett's positive cases for the subgroups with stromal enhancement and worse prognosis. These data need to be interpreted with care due to the limited numbers in our study and the incomplete data regarding prevalence of Barrett's esophagus. The significantly higher prevalence of Barrett's esophagus is in line with the results of the pathway analysis being suggestive of an influence of bile exposure as well as visceral adipocytes (as seen in obesity) playing a relevant role, both risk factors also relevant for Barrett's metaplasia and its progression.

Our study was not designed to develop a prognostic predictor panel. Explorative analysis of the available clinical data showed a modest prognostic effect that we interpret rather as proof‐of‐principle data supporting the biological relevance of our subtypes, rather than being of robust prognostic value when compared to other studies.^35^, ^36^ It is encouraging that our classification is also supported by the results from further independent datasets given that these comprised RNA‐Seq data or were generated on Affymetrix‐based platforms, whereas we used Illumina. Two of these cohorts comprised mainly cancers from Asian populations resulting in a different genetic background and different exposure to risk factors when compared to the Western patients of our primary cohort.^11^, ^12^ Although there seems to be some overlap between our new subgroups and the previously published classifications, study objectives, methods and design differed from our approach.

Interestingly, Kim and colleagues published data on a cohort of 64 patients with EAC, also demonstrating three subgroups when applying nonsupervised clustering on array‐based transcriptome data.^37^ They also demonstrated an association of their subgroup with prognosis. The gene list that served as the foundation for the subgroup assignment is not disclosed so comparison to our groups is limited. Furthermore, the target genes used for subgroup validation were selected based on Cox regression analysis and prognostic relevance whereas we aimed at selection based on biological dominance in the principal component analysis. It requires further prospective validation if our markers or the ones described by others before are useful for clinical application, and if so in which setting (e.g., as a prognostic marker, for treatment assignment or for individual preneoplastic risk assignment). We acknowledge that the results regarding different prognostic outcome for each group were not consistent across all individual validation datasets, but, most importantly, the three molecular subtypes were confirmed for all four validation cohorts, independent from the ethnic origin of the respective cohorts and the platform used for expression analysis.

The staining results in our cohort further support the transcriptome analysis. Some of the immunohistochemical markers have also been previously tested in malignant and premalignant stages of colorectal and gastroesophageal cancers.^38^, ^39^ The combination of CDH17 and CLDN18, for example, has been confirmed as being predictive for nodal involvement and poor prognosis in gastric adenocarcinomas.^40^ CLDN18 is a dominant marker in our poor prognosis Group 1 and CDH17 is characteristic for Group 2 which shows intermediate outcome in our primary cohort, but poor prognosis in some of the validation cohort. Some of our target genes have also been reported to be relevant for subtypes of pancreatic and right‐sided colorectal cancer, suggesting that similar mechanisms such as exposure to small bowel content (including bile and pancreatic enzymes) might be involved in carcinogenesis of gastroenteropancreatic tumors.^38^, ^41^ Similarly, dysregulation of specific transcription factors in Barrett's esophagus have been reported to be comparable to gene signals seen in normal colonic mucosa.^42^ Of note is the high expression of SULF1 in the two groups with poorer prognosis indicating again the relevance of stromal activation as poor prognostic factor. Saadi *et al*. demonstrated previously that there is a stage‐dependent stromal signature in Barrett's metaplasia, dysplasia and EAC that is associated with prognosis.^43^ While the aim of the previous study was the selection of a gene panel with optimal prognostic properties in the present study we aimed to an understanding of the biological background of the newly identified subtypes. This paves the way for further work to determine clinical significance.

In summary, our data show that the transcriptomic profiles of GEJ tumors reflect distinct molecular subgroups of intestinal type gastro‐esophageal adenocarcinomas indicative of cell biological function which is independent of anatomical location. Further understanding the biology of these subtypes will help to refine efforts for individualized targeted treatment as well as strategies for early detection and prevention.

## Author contributions

Study design: Bornschein J, under supervision from Fitzgerald RC and Malfertheiner P. Article drafting: Bornschein J, Wernisch L, Secrier M and Fitzgerald RC. Biostatistical analysis of the expression data: Bornschein J under guidance from Wernisch L and Newton R, and in cooperation with Perner J. IHC analysis: Bornschein J and Miremadi A. Biological samples process: MacRae S. Quality control of the histopathological data: O'Donovan M. Raw data processing and normalization processes of the expression data: Menon S and Eldridge MD. Whole genome sequencing data analysis: Secrier M, Bower L and Eldridge MD. RNA sequencing dataset curated and process: Devonshire G. Quality control of the clinical data: Bornschein J, Cheah C, and Turkington R. Sample acquisition contribution: Selgrad M and Venerito M. Funding for the study was obtained from Fitzgerald RC who takes responsibility for the data integrity.

## Supporting information


**Appendix S1**: Supporting Information

**Figure S1** Schematic overview on the sample collection and processing. Radiation‐ and chemotherapy‐naïve samples of gastroesophageal tumors have been prospectively collected before retrospective selection of samples with unequivocal allocation of the tumor location, with special focus on the gastroesophageal junction. Several checkpoints have been included to ensure quality of RNA and DNA before application of the Illumina HT v4.0 beadchip transcript array and whole‐genome sequencing.

**Figure S2** Output of the *mclust* algorithm for optimal group selection. The top panels indicate the geometrical distribution of the gene expression data for a different number of groups, with the higher number indicating a better fit of the respective distribution model, whereas the bottom panels show the corresponding principal component plots. The results for the core cohort of 61 GEJ cancers is shown in (a), whereas (b) shows the group including 23 further non‐junctional cancers. In both cases, a three group solution demonstrated the best results.

**Figure S3** Comparison of genomic data on a subcohort of the study population. Panel (a) shows the comparison of general genomic features such as total numbers of SNVs, tumor ploidy, total number of LOH or aberrant segments, and total number of amplified and deleted segments. There was no statistically significant difference between the groups. Panel (b) shows the distribution of each of the dominant mutational signatures within each group. The key signatures have been published before,^8^ but none of these could be seen clearly enriched in any of the groups. The type and accumulation of mutations affecting DNA damage repair (DDR) pathways are displayed in (c), where for each DDR category the percentage of samples within each subgroup with defects (nonsynonymous mutations/indels) in the respective pathway is highlighted.

**Figure S4 Grouping according to the mutational signature subtype.**
The barcharts illustrate the grouping according to the dominant mutational signature subtype as previously published by Secrier *et al*. in the exemplary subcohort with whole‐genome sequencing data. Group 3, the group with the best prognostic outcome, was enriched for the “DDR impaired” group as indicated in main data (a). This was not statistically significant. In the OCCAMS validation cohort (b) there was enrichment for “DDR impaired” tumors in Group 2, again without reaching statistical significance.

**Figure S5** Prognostic outcome in independent tumor cohorts when stratified by the 67‐gene panel. Displayed are Kaplan–Meier curves for overall survival comparing the newly identified tumor subtypes in independent cohorts of patients with other tumor entities. For none of the additional carcinoma entities displayed in the bottom row (colorectal, breast and lung) a significantly different outcome could be confirmed if the cohorts have been stratified according to the 67‐gene panel.

**Figure S6 Relative expression of phenotypically relevant genes.**
The boxplots indicate the relative gene expression of *CD8A*, *GZMB*, and *ASCL2. CD8A* and *GZMB* indicate T‐cell activation and are more highly expressed in Group 3, which is enriched for immune response pathways. *ASCL2* is an intestinal stemness marker and is dominant in Group 2 which shows features of metaplastic processes of the intestinal type.

**Table S1** Differential gene expression between cancers of different Siewert type, and between junctional and nonjunctional cancers on pairwise comparison as assessed by *limma*

**Table S2** Differential gene expression between the three groups on pairwise comparison as assessed by *limma*

**Table S3** Differential gene expression between the three cancer groups and noncancer controls on pairwise comparison as assessed by *limma*

**Table S4** Cut‐off levels for positive immune‐reactivity score for each marker and results for each subtype

**Table S5** Pathway analysis of the three groups based on generally applicable gene set enrichment for pathway analysis (*gage*) and KEGG as well as *Gene Ontology* terms

**Table S6** Ingenuity Pathway Analysis (IPA®) of the three groups

**Table S7** Demographic and clinicopathological data of the study cohort (*n* = 107)

## Appendix 1

**Oesophageal Cancer Clinical and Molecular Stratification (OCCAMS) Consortium Members List**

Ayesha Noorani^1^, Rachael Fels Elliott^1^, Paul A.W. Edwards^1,2^, Nicola Grehan^1^, Barbara Nutzinger^1^, Jason Crawte^1^, Hamza Chettouh^1^, Gianmarco Contino^1^, Xiaodun Li^1^, Eleanor Gregson^1^, Sebastian Zeki^1^, Rachel de la Rue^1^, Shalini Malhotra^1,3^, Simon Tavaré^2^, Andy G. Lynch^2^, Mike L. Smith^2^, Jim Davies^5^, Charles Crichton^5^, Nick Carroll^6^, Peter Safranek^6^, Andrew Hindmarsh^6^, Vijayendran Sujendran^6^, Stephen J. Hayes^7,14^, Yeng Ang^7,8,29^, Shaun R. Preston^9^, Sarah Oakes^9^, Izhar Bagwan^9^, Vicki Save^10^, Richard J.E. Skipworth^10^, Ted R. Hupp^10^, J. Robert O'Neill^10,23^, Olga Tucker^11,33^, Andrew Beggs^11,28^, Philippe Taniere^11^, Sonia Puig^11^, Timothy J. Underwood^12,13^, Fergus Noble^12^, Jack Owsley^12^, Hugh Barr^15^, Neil Shepherd^15^, Oliver Old^15^, Jesper Lagergren^16,25^, James Gossage^16,24^, Andrew Davies^16,24,^ Fuju Chang^16,24^, Janine Zylstra^16,24^, Vicky Goh^24^, Francesca D. Ciccarelli^24^, Grant Sanders^17^, Richard Berrisford^17^, Catherine Harden^17^, David Bunting^17^, Mike Lewis^18^, Ed Cheong^18^, Bhaskar Kumar^18^, Simon L. Parsons^19^, Irshad Soomro^19^, Philip Kaye^19^, John Saunders^19^, Laurence Lovat^20^, Rehan Haidry^20^, Victor Eneh^20^, Laszlo Igali^21^, Michael Scott^22^, Shamila Sothi^26^, Sari Suortamo^26^, Suzy Lishman^27^, George B. Hanna^31^, Christopher J. Peters^31^, Anna Grabowska^32^

^1^Medical Research Council Cancer Unit, Hutchison/Medical Research Council Research Centre, University of Cambridge, Cambridge, UK; ^2^Cancer Research UK Cambridge Institute, University of Cambridge, Cambridge, UK; ^3^Department of Histopathology, Addenbrooke's Hospital, Cambridge, UK; ^4^Oxford ComLab, University of Oxford, UK, OX1 2JD; ^5^Department of Computer Science, University of Oxford, UK, OX1 3QD; ^6^Cambridge University Hospitals NHS Foundation Trust, Cambridge, UK, CB2 0QQ; ^7^Salford Royal NHS Foundation Trust, Salford, UK, M6 8HD; ^8^Wigan and Leigh NHS Foundation Trust, Wigan, Manchester, UK, WN1 2NN; ^9^Royal Surrey County Hospital NHS Foundation Trust, Guildford, UK, GU2 7XX; ^10^Edinburgh Royal Infirmary, Edinburgh, UK, EH16 4SA; ^11^University Hospitals Birmingham NHS Foundation Trust, Birmingham, UK, B15 2GW; ^12^University Hospital Southampton NHS Foundation Trust, Southampton, UK, SO16 6YD; ^13^Cancer Sciences Division, University of Southampton, Southampton, UK, SO17 1BJ; ^14^Faculty of Medical and Human Sciences, University of Manchester, UK, M13 9PL; ^15^Gloucester Royal Hospital, Gloucester, UK, GL1 3NN; ^16^St Thomas's Hospital, London, UK, SE1 7EH; ^17^Plymouth Hospitals NHS Trust, Plymouth, UK, PL6 8DH; ^18^Norfolk and Norwich University Hospital NHS Foundation Trust, Norwich, UK, NR4 7UY; ^19^Nottingham University Hospitals NHS Trust, Nottingham, UK, NG7 2UH; ^20^University College London, London, UK, WC1E 6BT; ^21^Norfolk and Waveney Cellular Pathology Network, Norwich, UK, NR4 7UY; ^22^Wythenshawe Hospital, Manchester, UK, M23 9LT; ^23^Edinburgh University, Edinburgh, UK, EH8 9YL; ^24^King's College London, London, UK, WC2R 2LS; ^25^Karolinska Institutet, Stockholm, Sweden, SE‐171 77; ^26^University Hospitals Coventry and Warwickshire NHS, Trust, Coventry, UK, CV2 2DX; ^27^Peterborough Hospitals NHS Trust, Peterborough City Hospital, Peterborough, UK, PE3 9GZ; ^28^Institute of Cancer and Genomic sciences, University of Birmingham, B15 2TT; ^29^GI science centre, University of Manchester, UK, M13 9PL; ^30^Queen's Medical Centre, University of Nottingham, Nottingham, UK, NG7 2UH; ^31^Imperial College NHS Trust, Imperial College London, UK, W2 1NY; ^32^Queen's Medical Centre, University of Nottingham, Nottingham, UK; ^33^Heart of England NHS Foundation Trust, Birmingham, UK, B9 5SS.
