# Supplementary material for: Transcriptomic profiling reveals three molecular phenotypes of adenocarcinoma at the gastroesophageal junction
Source: Int J Cancer. 2019 May 17;145(12):3389–401. doi: 10.1002/ijc.32384 (PMC6851674; doi:10.1002/ijc.32384)
Supplement: Supplementary file 1 — Appendix S1: Supporting Information [file IJC-145-3389-s001.docx]

SUPPLEMENTARY METHODS

1. **Raw data processing**

Raw intensity data from the array scanner was processed using the BASH^1^ and HULK algorithms as implemented in the *beadarray* package^2^. Log2 transformation and quantile normalisation of the data was performed. After exclusion of non-cancer controls (only batch 2), batches 1 and 2 were merged using *inSilicoMerging*, applying *GENENORM* for batch correction, resulting in a random distribution of samples of each batch within the merged dataset^3^. At this point, technical replicates that have been used as internal controls were also excluded for further analysis to avoid cohort selection bias.

1. **Definition of intrinsic subtypes and pathway analysis**

Selected gene probes that were labelled as “good” or “perfect” match with the respective reference sequence for each gene were used according to the annotation provided by Illumina, leaving n=34,476 probes. *Limma* was used in R for differential gene expression analysis^4^. To assess the sensitivity of this method to pick up differentially expressed genes in our cohort with small sample size, we performed a simulation, in which 100 genes were randomly picked and one of their Siewert group values artificially increased with a certain effect size (fractions of overall SD). Sensitivity curves at each simulated level of effect size demonstrated adequate power to pick up respective differentially expressed genes at an FDR threshold of 0.05. The script for differential gene expression analysis was identical to the one applied in the original Siewert comparison.

For the explorative analysis for intrinsic subgroups, the 1000 most variable probes within the cohort were selected, before *mclust* was applied^5^. For the whole cohort (N=107) *mclust* suggested a 2-group solution based on a VVI distribution model. Differential gene expression analysis (*limma*^4^) of these two groups revealed for the smaller group of 23 tumors strong enrichment for genes that are specific in the regulation and maintenance of squamous epithelium. Critical review of the H&E sections of the tissue blocks which were used for RNA extraction suggested a high likelihood of contamination with tumor-adjacent normal squamous epithelium of the esophagus in proximal cancers despite careful micro-dissection. To exclude a bias by this factor, these 23 samples were excluded for the core analysis, but used later for validation of the selected gene panel.

We repeated the steps above (selection of the 1000 most variable probes, *mclust* based groups assignment and differential gene expression in *limma*) in parallel for the remaining 84 cancers as well as separately for the 61 remaining GEJ tumors. For both approaches, *mclust* suggested a three-group solution based on a diagonal distribution model (VEI model) applying Bayesian information criteria (BIC). Group by group comparisons revealed differentially regulated genes as listed in **Supplementary Table 2**. Since the expression profiles have been used twice in the statistical analysis, to define the clusters as well as to calculate *p*-values of differential expression between these clusters, the p-values lost their original statistical meaning and we therefore term the resulting values as p-scores. *P*-scores are still useful to select a gene panel which in the following proved to be useful for predictive purpose. For further selection of a representative gene panel we therefore applied a strict cut-off of a *p*-score <0.0001 and a minimal log-fold change of 2.0. Only genes that were represented in both approaches (n=84 and n=61) were selected, resulting in a 67 genes panel.

We performed a cross validation for the group prediction using the primary cohort data based on the gene panel in the following way. We trained the gene panel on randomly selected 52 samples among the 104 for group prediction and applied the group prediction on the other 52 samples in 1000 iterations. In each iteration, we compared the predicted group with the original group: the two larger groups were predicted correctly in all and the small group in 93% of the iterations. This indicates that the clustering structure is pronounced enough, and the prediction based on the gene panel reliable enough to enable prediction of group membership for new samples.

Gene-set based pathway analysis was performed based on KEGG and Gene Ontology (GO) terms using *gage* in R^6^. For further comprehensive analysis, also *Ingenuity® Pathway Analysis* (IPA®, QIAGEN Redwood City, [www.qiagen.com/ingenuity](http://www.qiagen.com/ingenuity)) was performed. Pathway analysis was also applied for batch 2 only to compare with non-cancer controls (separate analysis for duodenal and gastric control samples).

For further validation of our results the 67-gene panel was applied using *mclust* on publicly available independent datasets, which comprised either oesophageal adenocarcinomas (BELFAST, E-MTAB-4666), or gastric cancers (SINGAPORE, GSE15459^7^; ACRG, GSE62254^8^), to reproduce the three groups defined in our primary cohort. In contrast to our Illumina beadchip data, the external datasets were generated on Affymetrix based platforms (BELFAST: customised Affymetrix-based platform; SINGAPORE, ACRG: Affymetrix - GPL570).

As a control for subgroup comparability among the datasets and platforms for each gene all probes were averaged to obtain a gene-specific expression matrix for each of the four cohorts. Each gene was assigned to the cluster wherein it had its highest expression. Therefore, in new datasets the genes define the respective clusters. A contingency table of all gene votes was then used to clarify which cluster corresponds to which gene vote. Please see the main text for further details on this analysis.

1. **Whole-genome sequencing analysis**

Whole-genome sequencing was performed on a tumor and matched normal sample for each patient in the cohort. Blood or normal squamous esophageal samples, distant by at least 5 cm from the tumor, were used as germline reference. A single library was created for each sample, and 100-bp paired-end sequencing was performed under contracts by Illumina and the Broad Institute to a typical depth of at least 50x for tumors and 30x for matched normals, with 94% of the known genome being sequenced to at least 8x coverage and achieving a Phred quality of at least 30 for at least 80% of mapping bases. Read sequences were mapped to the human reference genome (GRCh37) using Burrows-Wheeler Alignment (BWA) 0.5.9^9^ and duplicates were marked and discarded using Picard 1.105 (http://broadinstitute.github.io/picard). As part of an extensive quality assurance process, quality control metrics and alignment statistics were computed on a per-lane basis. The *FastQC* package was used to assess the quality score distribution of the sequencing reads and perform trimming if necessary. Samples were examined for potential microsatellite instability using *MSIsensor*^10^, and no clear evidence for such cases was found in the cohort.

**Somatic mutation and indel calling**

Somatic mutations and indels were called using Strelka 1.0.13^11^. SNVs were filtered as described in the **table** below. Functional annotation of the resulting variants was performed using Variant Effect Predictor (VEP release 75)^12^.

**Copy number and loss of heterozygosity analysis**

Absolute copy number after correction for estimated normal-cell contamination was called with ASCAT-NGS v2.1^13^, using read counts at germline heterozygous positions estimated by GATK 3.2-2^14^. Regions with an absolute copy number ≥2-fold the average ploidy for the sample were deemed as amplified, while deleted regions were defined as segments with an absolute copy number ≤ 0.5-fold the average ploidy. Loss of heterozygosity (LOH) was defined as ASCAT-estimated minor allele copy number of 0.

**Mutational signature analysis**

Mutational signatures were identified using the methodology described by Alexandrov *et al.*^15^. Before running the software, common variants in the 1000 genomes database^16^ appearing in at least 0.5% of the population were removed. The optimal number of signatures in the dataset was chosen to balance the signature stability against the Frobenius reconstruction error.

The assignment of patients into the 3 mutational signature subtypes defined in Secrier, Li *et al.*^17^ was performed based on the dominant signature in each sample.

**DNA damage repair (DDR) analysis**

To assess the alterations in DNA damage-related pathways in our cohort, we performed an analysis similar to the one described by Pearl *et al.*^18^ and further detailed in Secrier & Li *et al.*^17^. Among the genes involved in defined DNA damage pathways as described in the paper, we catalogued those that harboured nonsynonymous somatic mutations/indels in our cohort. We then compared the number of samples with such alterations in 16 main pathways among the defined expression-based groups.

1. **Immunohistochemistry Analysis**

For immunohistochemistry the following antibodies and respective dilutions have been used: mucin-5AC (MUC5AC: ab3649, Abcam; 1:200 dilution), cathepsin-E (CTSE: HPA012940, Atlas; 1:100), claudin-18 (CLDN18: NBP2-32002, Novus; 1:200), caudal-type homeobox transcription factor 1 (CDX1: ab188072, Abcam; 1:50), cadherin-17 (CDH17: HPA023616, Sigma-Aldrich; 1:1000), chemokine (C-X-C-motif) ligand-10 (CXCL10/IP10: (ab-9807, Abcam; 1:200), sulfatase-1 (SULF1: NBP2-31584, Novus; 1:200), trefoil factor 2 (TFF2: HPA036705, Atlas; 1:100), indoleamine-2,3-dioxygenase 1 (IDO1: ab55305, Abcam; 1:1000). Incubation time was 20 minutes for all markers. Antigen retrieval was performed at pH=6 for all markers but SULF1 for which pH=9 was applied.

Staining was scored in concordance with the modified immune-reactivity score by Remmele and Stegner^19^. For CDX1 this score has been modified for the assessment of the nuclear staining, for CLDN18 and CDH17 for assessment of the membranous staining.

To assess validity of the staining, the IRS for each marker were correlated with the respective gene expression values generated by the transcript array. The immunostaining score showed a good correlation with the relative mRNA expression values for MUC5AC (*r*=0.542, *p*=0.002), CTSE (*r*=0.480, *p*=0.008), TFF2 (*r*=0.605, *p*=0.001) and cytosolic CDH17 (*r*=0.527, *p*=0.003). Correlation for the other markers failed to reach the threshold of statistical significance which might be due to the small numbers and selective assessment of e.g. membranous staining or of specific stromal cellular components (i.e. immune cells, fibroblasts).

1. **Statistics**

All statistical tests comparing whole-genome sequencing data metrics were performed using a Wilcoxon rank-sum test or ANOVA (for continuous data), and a Fisher exact test or Chi-square test (for count data). For comparison of the IHC scores, the Kruskal-Wallis test was applied for general comparison of raw reactivity scores and Fisher’s exact test for comparison of the overall expression status (SPSS 23.0, IBM SPSS Statistics for Windows, Armonk, NY: IBM Corp.).

**Table: Summary of filters applied to single nucleotide variant (SNV) calls from Strelka.** Filters were created, and thresholds chosen by assessing kernel density plots of true and false positive SNV calls for a medulloblastoma *International Cancer Genome Consortium* (ICGC) benchmark dataset.

| **Filter** | **Description** |
| --- | --- |
| DistanceToAlignmentEndMedian | The median shortest distance of the variant position within the read to either aligned end is less than 10 |
| DistanceToAlignmentEndMAD | The median absolute deviation of the shortest distance of the variant position within the read to either aligned end is less than 3 |
| LowMapQual | The proportion of reads at the variant position with low mapping quality (less than 1) is greater than 10% |
| MapQualDiffMedian | The difference in the median mapping quality of variant reads (in the tumour) and reference reads (in the normal) is greater than 5 |
| VariantMapQualMedian | The median mapping quality of variant reads is less than 40 |
| VariantBaseQualMedian | The median base quality at the variant position of variant reads is less than 30 |
| VariantAlleleCount | The number of variant-supporting reads in the tumour is less than 4 |
| VariantAlleleCountControl | The number of variant-supporting reads in the normal is greater than 1 |
| StrandBias | The strand bias for variant reads covering the variant position, i.e. the fraction of reads in either direction, is less than 0.02, unless the strand bias for all reads is also less than 0.02. |
| Repeat | The length of repetitive sequence adjacent to the variant position, where repeats can be 1-, 2-, 3-, or 4-mers, is 12 or more |
| SNVCluster50 | The largest number of variant positions within any 50 base pair window surrounding, but excluding, the variant position is greater than 2; variant positions are those in which the number of alternate allele is supported by at least 2 reads and at least 5% of all reads covering that position. |
| SNVCluster100 | The largest number of variant positions within any 100 base pair window surrounding, but excluding, the variant position is greater than 4; variant positions are those in which the number of alternate allele is supported by at least 2 reads and at least 5% of all reads covering that position. |

**References**

1. Cairns JM, Dunning MJ, Ritchie ME, Russell R, Lynch AG. BASH: a tool for managing BeadArray spatial artefacts. Bioinformatics 2008;24:2921–2.

2. Dunning MJ, Smith ML, Ritchie ME, Tavare S. beadarray: R classes and methods for Illumina bead-based data. Bioinformatics 2007;23:2183–4.

3. Taminau J, Meganck S, Lazar C, Steenhoff D, Coletta A, Molter C, Duque R, de Schaetzen V, Weiss Solís DY, Bersini H, Nowé A. Unlocking the potential of publicly available microarray data using inSilicoDb and inSilicoMerging R/Bioconductor packages. BMC Bioinformatics 2012;13:335.

4. Ritchie ME, Phipson B, Wu D, Hu Y, Law CW, Shi W, Smyth GK. limma powers differential expression analyses for RNA-sequencing and microarray studies. Nucleic Acids Res 2015;43:e47.

5. Yeung KY, Fraley C, Murua A, Raftery AE, Ruzzo WL. Model-based clustering and data transformations for gene expression data. Bioinformatics 2001;17:977–87.

6. Luo W, Friedman MS, Shedden K, Hankenson KD, Woolf PJ. GAGE: generally applicable gene set enrichment for pathway analysis. BMC Bioinformatics 2009;10:161.

7. Lei Z, Tan IB, Das K, Deng N, Zouridis H, Pattison S, Chua C, Feng Z, Guan YK, Ooi CH, Ivanova T, Zhang S, et al. Identification of molecular subtypes of gastric cancer with different responses to PI3-kinase inhibitors and 5-fluorouracil. Gastroenterology 2013;145:554–65.

8. Cristescu R, Lee J, Nebozhyn M, Kim K-M, Ting JC, Wong SS, Liu J, Yue YG, Wang J, Yu K, Ye XS, Do I-G, et al. Molecular analysis of gastric cancer identifies subtypes associated with distinct clinical outcomes. Nat Med 2015;21:449–56.

9. Li H, Durbin R. Fast and accurate short read alignment with Burrows-Wheeler transform. Bioinformatics 2009;25:1754–60.

10. Niu B, Ye K, Zhang Q, Lu C, Xie M, McLellan MD, Wendl MC, Ding L. MSIsensor: microsatellite instability detection using paired tumor-normal sequence data. Bioinformatics 2014;30:1015–6.

11. Saunders CT, Wong WSW, Swamy S, Becq J, Murray LJ, Cheetham RK. Strelka: accurate somatic small-variant calling from sequenced tumor-normal sample pairs. Bioinformatics 2012;28:1811–7.

12. McLaren W, Pritchard B, Rios D, Chen Y, Flicek P, Cunningham F. Deriving the consequences of genomic variants with the Ensembl API and SNP Effect Predictor. Bioinformatics 2010;26:2069–70.

13. Van Loo P, Nordgard SH, Lingjaerde OC, Russnes HG, Rye IH, Sun W, Weigman VJ, Marynen P, Zetterberg A, Naume B, Perou CM, Borresen-Dale A-L, et al. Allele-specific copy number analysis of tumors. Proc Natl Acad Sci U S A 2010;107:16910–5.

14. McKenna A, Hanna M, Banks E, Sivachenko A, Cibulskis K, Kernytsky A, Garimella K, Altshuler D, Gabriel S, Daly M, DePristo MA. The Genome Analysis Toolkit: a MapReduce framework for analyzing next-generation DNA sequencing data. Genome Res 2010;20:1297–303.

15. Alexandrov LB, Nik-Zainal S, Wedge DC, Aparicio SAJR, Behjati S, Biankin A V, Bignell GR, Bolli N, Borg A, Borresen-Dale A-L, Boyault S, Burkhardt B, et al. Signatures of mutational processes in human cancer. Nature 2013;500:415–21.

16. 1000 Genomes Project Consortium, Auton A, Brooks LD, Durbin RM, Garrison EP, Kang HM, Korbel JO, Marchini JL, McCarthy S, McVean GA, Abecasis GR. A global reference for human genetic variation. Nature 2015;526:68–74.

17. Secrier M, Li X, de Silva N, Eldridge MD, Contino G, Bornschein J, MacRae S, Grehan N, O’Donovan M, Miremadi A, Yang T-P, Bower L, et al. Mutational signatures in esophageal adenocarcinoma define etiologically distinct subgroups with therapeutic relevance. Nat Genet 2016;48:1131–41.

18. Pearl LH, Schierz AC, Ward SE, Al-Lazikani B, Pearl FMG. Therapeutic opportunities within the DNA damage response. Nat Rev Cancer 2015;15:166–80.

19. Remmele W, Stegner HE. [Recommendation for uniform definition of an immunoreactive score (IRS) for immunohistochemical estrogen receptor detection (ER-ICA) in breast cancer tissue]. Pathologe 1987;8:138–40.
