# Supplementary figures and images for: Transcriptomic profiling reveals three molecular phenotypes of adenocarcinoma at the gastroesophageal junction
Source: Int J Cancer. 2019 May 17;145(12):3389–401. doi: 10.1002/ijc.32384 (PMC6851674; doi:10.1002/ijc.32384)

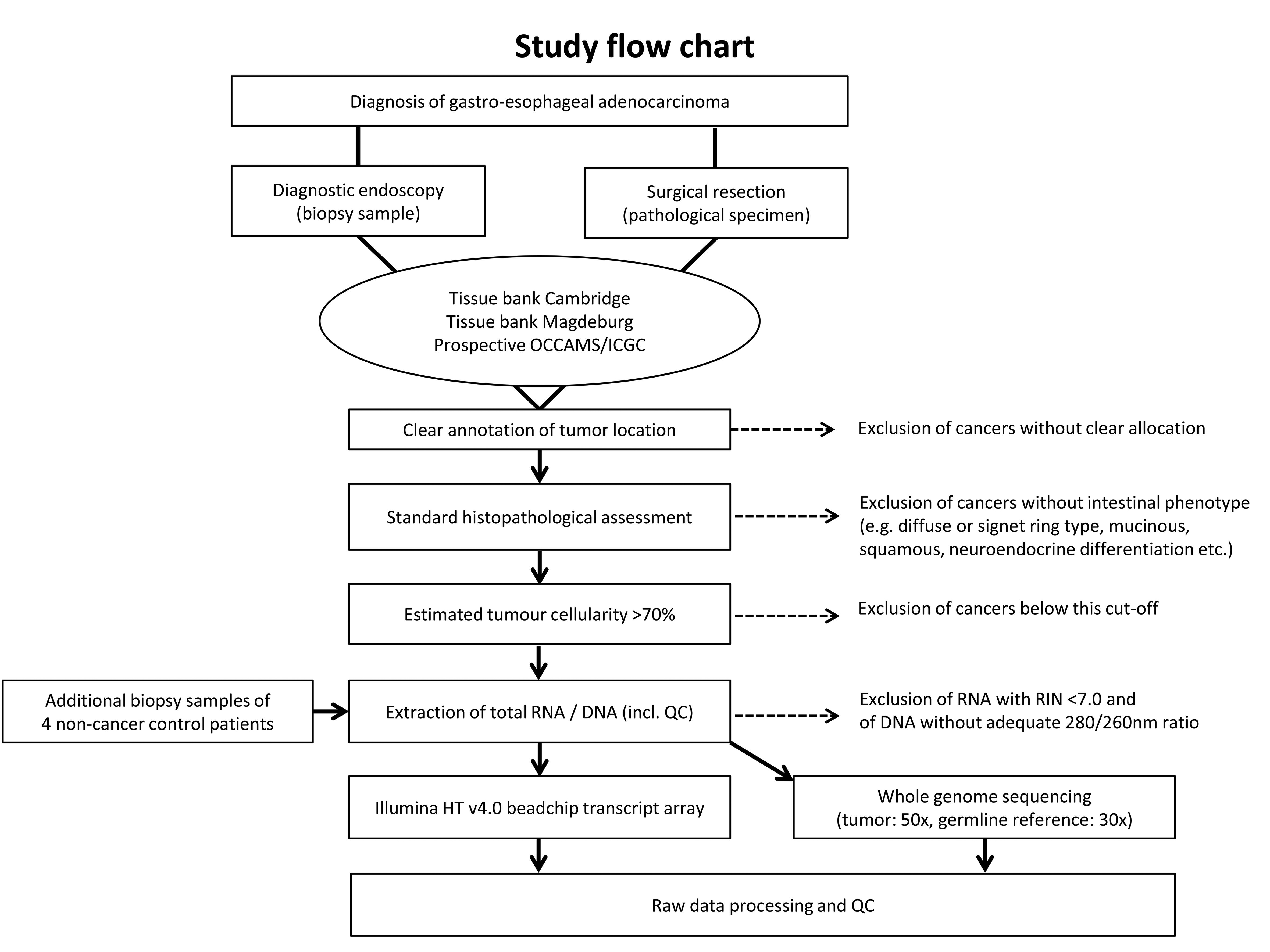

Supplement: Supplementary file 2 — Figure S1 Schematic overview on the sample collection and processing. Radiation‐ and chemotherapy‐naïve samples of gastroesophageal tumors have been prospectively collected before retrospective selection of samples with unequivocal allocation of the tumor location, with special focus on the gastroesophageal junction. Several checkpoints have been included to ensure quality of RNA and DNA before application of the Illumina HT v4.0 beadchip transcript array and whole‐genome sequencing. [file IJC-145-3389-s002.tif]

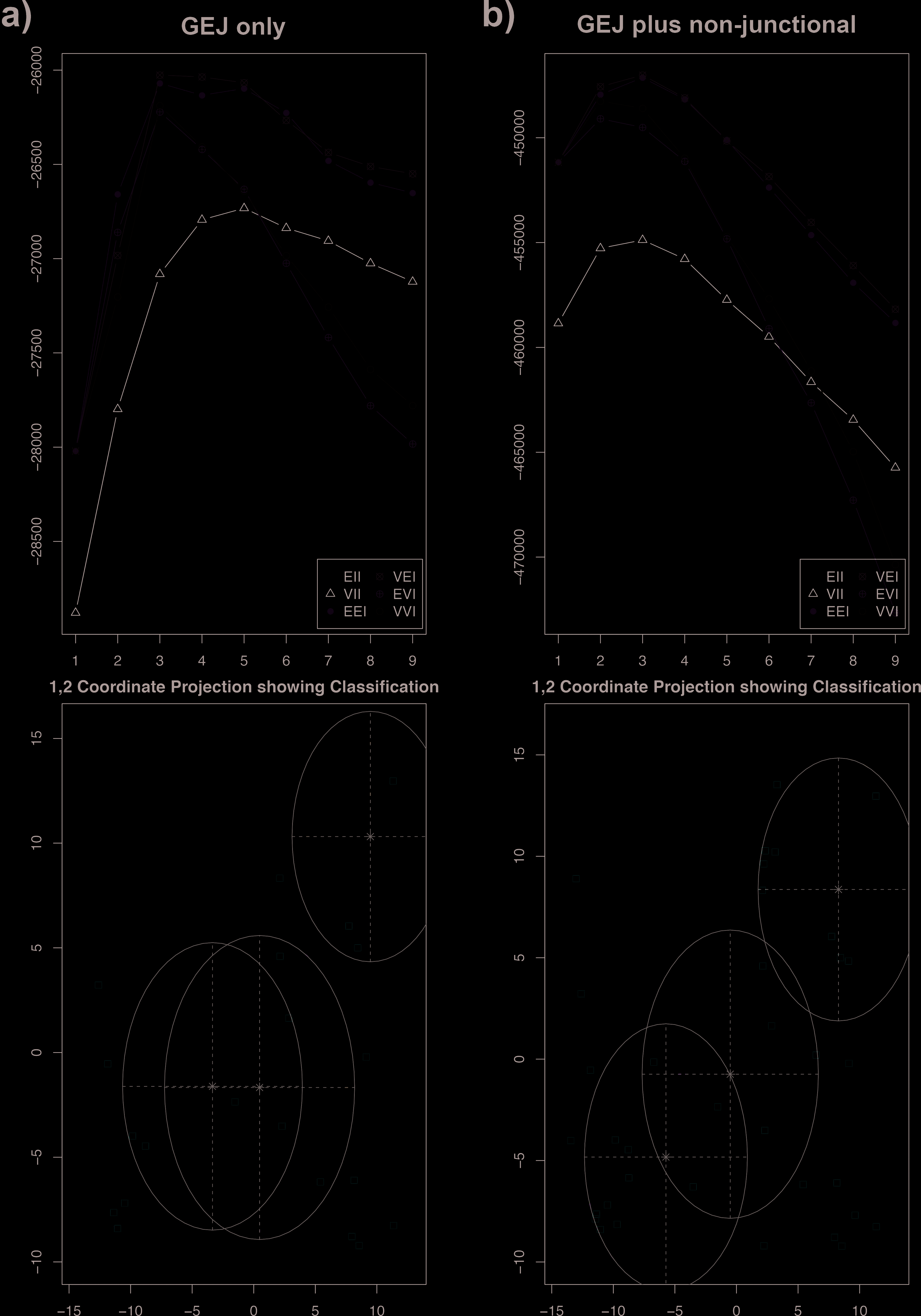

Supplement: Supplementary file 3 — Figure S2 Output of the mclust algorithm for optimal group selection. The top panels indicate the geometrical distribution of the gene expression data for a different number of groups, with the higher number indicating a better fit of the respective distribution model, whereas the bottom panels show the corresponding principal component plots. The results for the core cohort of 61 GEJ cancers is shown in (a), whereas (b) shows the group including 23 further non‐junctional cancers. In both cases, a three group solution demonstrated the best results. [file IJC-145-3389-s003.tif]

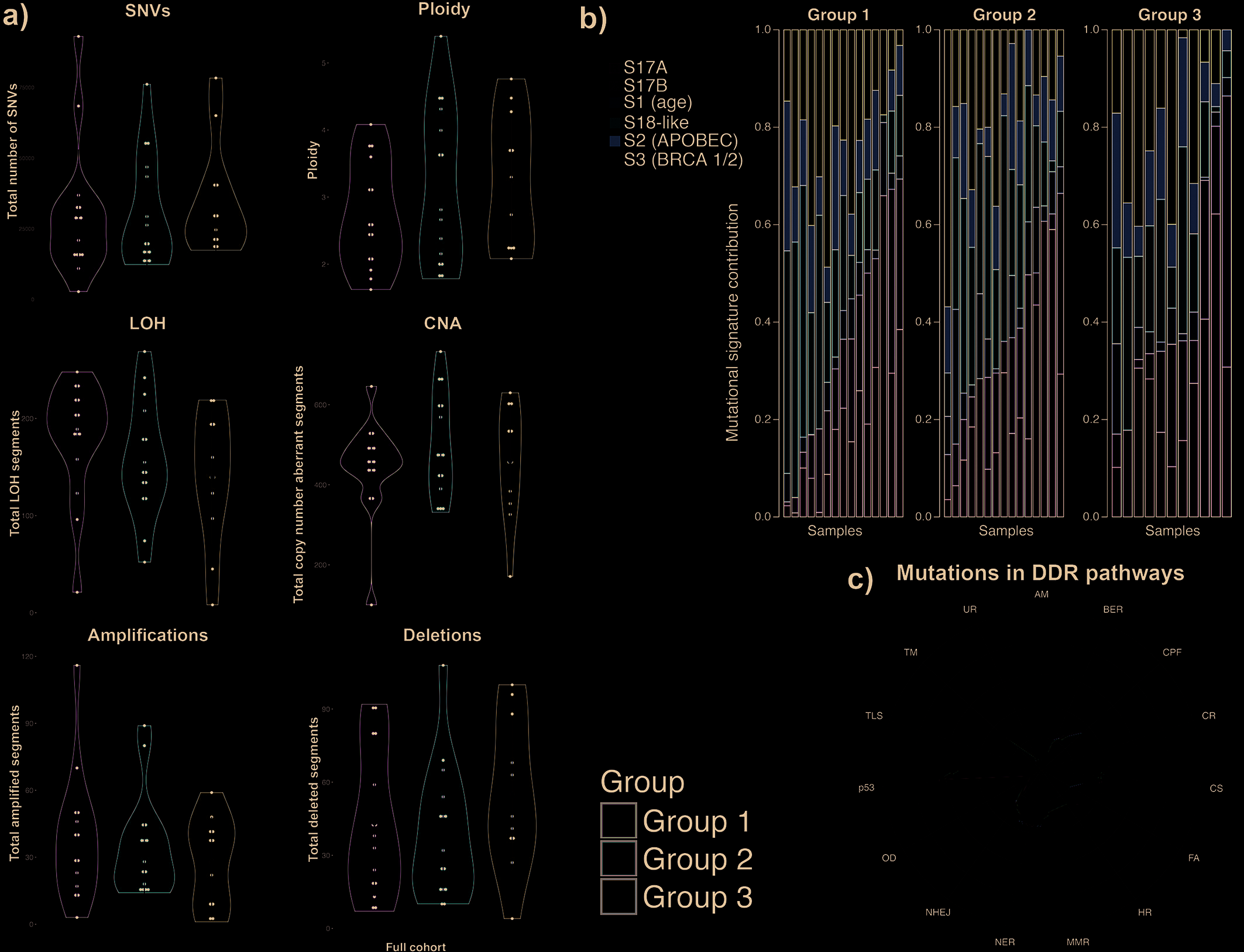

Supplement: Supplementary file 4 — Figure S3 Comparison of genomic data on a subcohort of the study population. Panel (a) shows the comparison of general genomic features such as total numbers of SNVs, tumor ploidy, total number of LOH or aberrant segments, and total number of amplified and deleted segments. There was no statistically significant difference between the groups. Panel (b) shows the distribution of each of the dominant mutational signatures within each group. The key signatures have been published before,8 but none of these could be seen clearly enriched in any of the groups. The type and accumulation of mutations affecting DNA damage repair (DDR) pathways are displayed in (c), where for each DDR category the percentage of samples within each subgroup with defects (nonsynonymous mutations/indels) in the respective pathway is highlighted. [file IJC-145-3389-s004.tif]

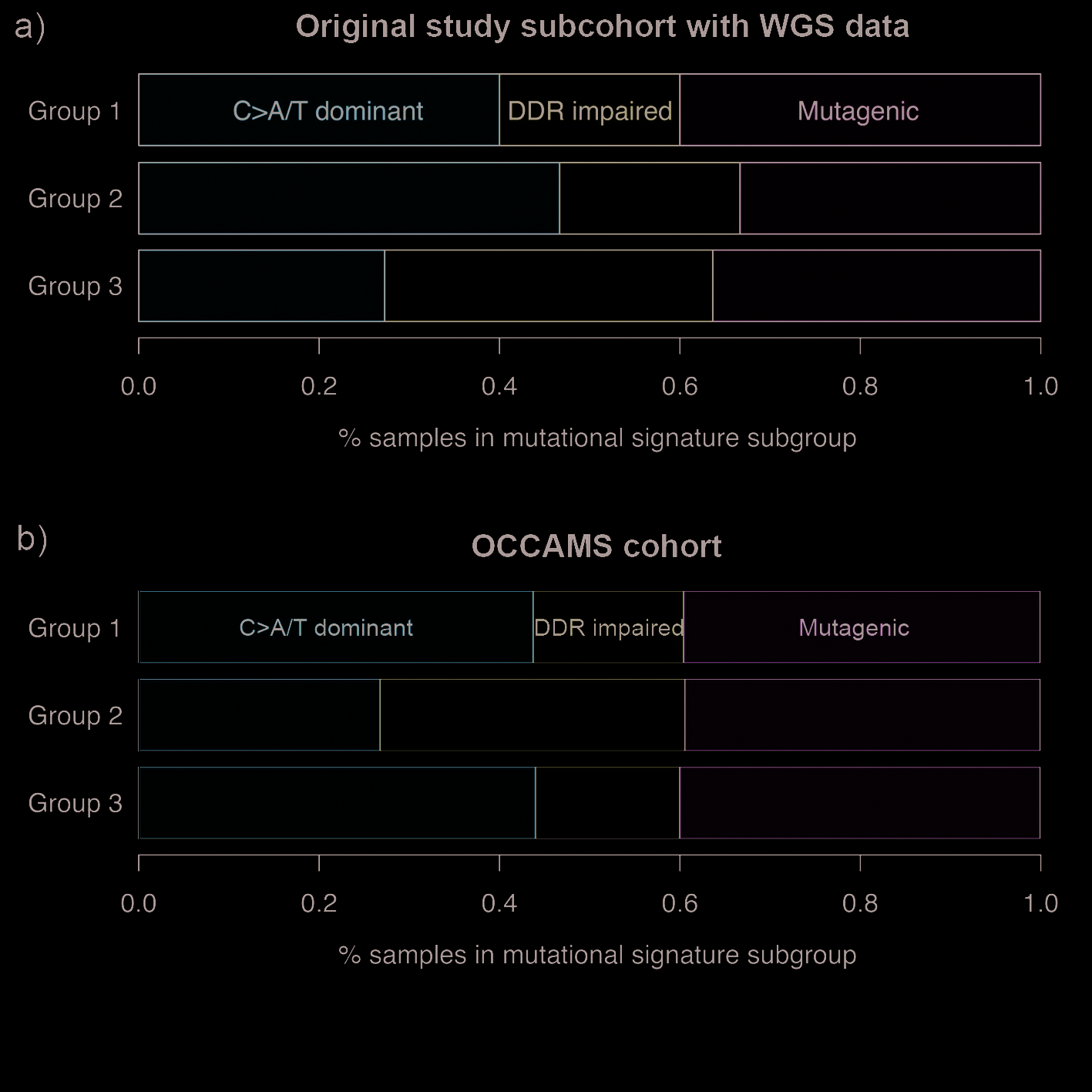

Supplement: Supplementary file 5 — Figure S4 Grouping according to the mutational signature subtype. The barcharts illustrate the grouping according to the dominant mutational signature subtype as previously published by Secrier et al. in the exemplary subcohort with whole‐genome sequencing data. Group 3, the group with the best prognostic outcome, was enriched for the “DDR impaired” group as indicated in main data (a). This was not statistically significant. In the OCCAMS validation cohort (b) there was enrichment for “DDR impaired” tumors in Group 2, again without reaching statistical significance. [file IJC-145-3389-s005.tif]

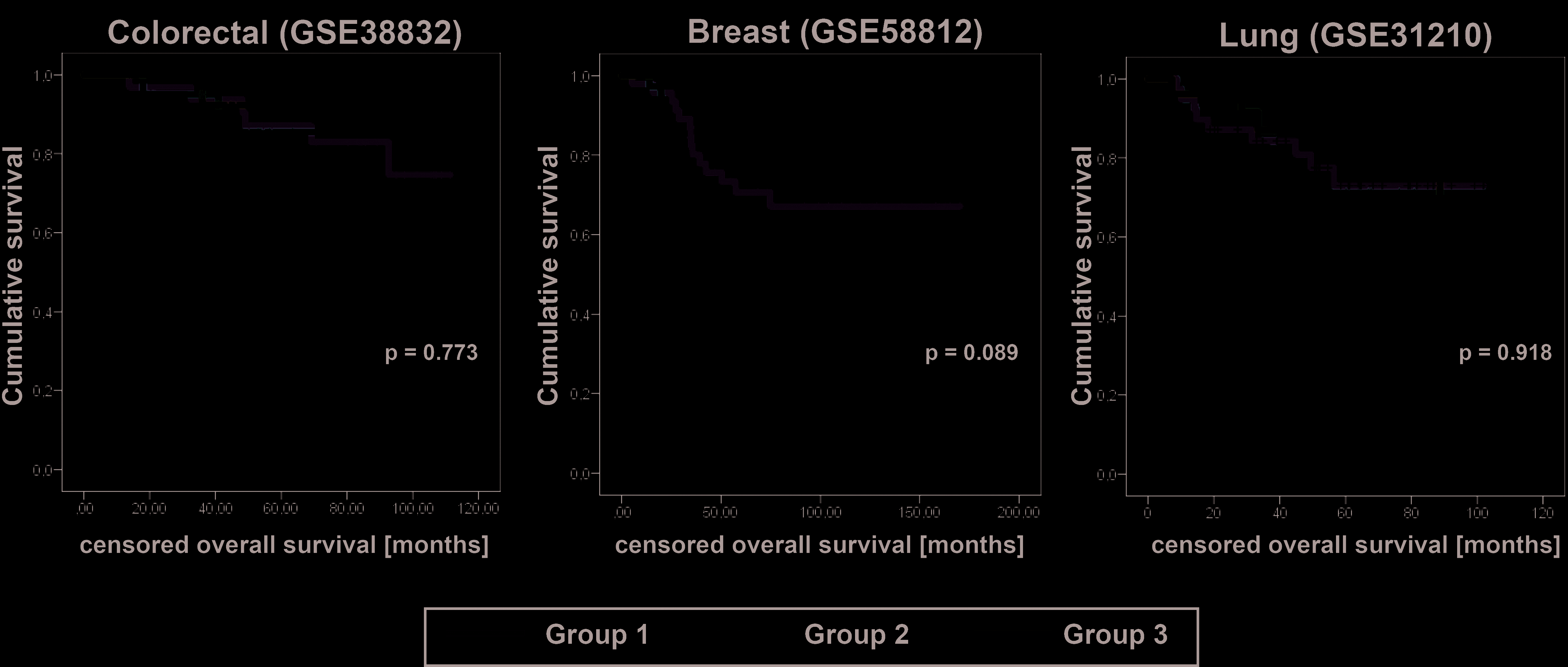

Supplement: Supplementary file 6 — Figure S5 Prognostic outcome in independent tumor cohorts when stratified by the 67‐gene panel. Displayed are Kaplan–Meier curves for overall survival comparing the newly identified tumor subtypes in independent cohorts of patients with other tumor entities. For none of the additional carcinoma entities displayed in the bottom row (colorectal, breast and lung) a significantly different outcome could be confirmed if the cohorts have been stratified according to the 67‐gene panel. [file IJC-145-3389-s006.tif]

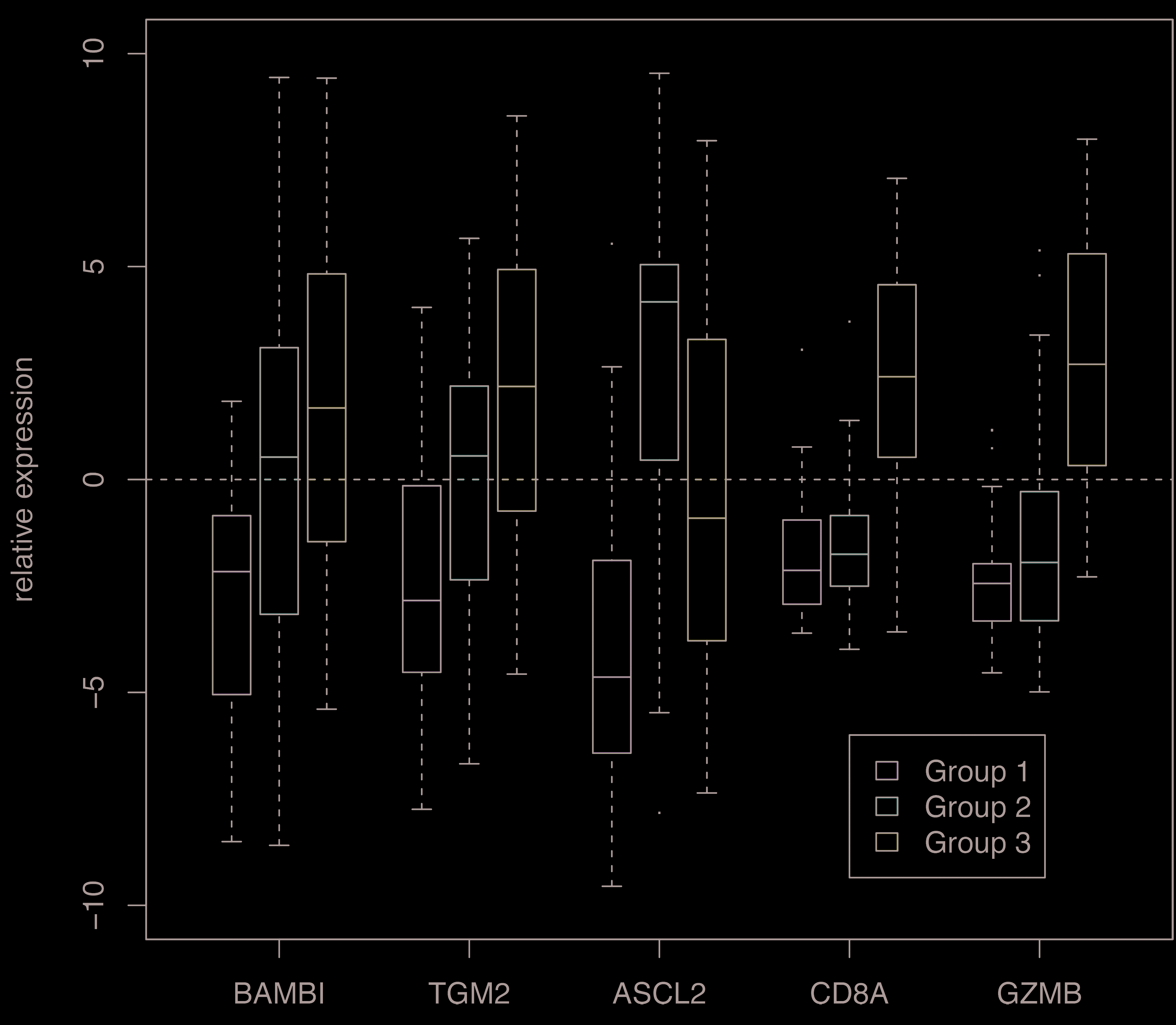

Supplement: Supplementary file 7 — Figure S6 Relative expression of phenotypically relevant genes. The boxplots indicate the relative gene expression of CD8A, GZMB, and ASCL2. CD8A and GZMB indicate T‐cell activation and are more highly expressed in Group 3, which is enriched for immune response pathways. ASCL2 is an intestinal stemness marker and is dominant in Group 2 which shows features of metaplastic processes of the intestinal type. [file IJC-145-3389-s007.tif]
