# Supplementary material for: Transcriptomic profiling reveals three molecular phenotypes of adenocarcinoma at the gastroesophageal junction
Source: Int J Cancer. 2019 May 17;145(12):3389–401. doi: 10.1002/ijc.32384 (PMC6851674; doi:10.1002/ijc.32384)
Supplement: Supplementary file 11 — Table S4 Cut‐off levels for positive immune‐reactivity score for each marker and results for each subtype [file IJC-145-3389-s011.docx]

**Supplementary Table 7: Median Immune-Reactivity Score (IRS*) for the selected markers in each sub-group.**

|  | Cellular compartment | **Group 1** | **Group 2** | **Group 3** | **p-value** |
| --- | --- | --- | --- | --- | --- |
| **MUC5AC** | cytoplasmatic | 20,0 | 5,0 | 2,5 | 0.233 |
| **TFF2** | cytoplasmatic | 5,0 | 2,0 | 1,0 | 0.071 |
| **CTSE** | cytoplasmatic | 14,0 | 2,5 | 7,5 | **0.047** |
| **CLDN18** | cytoplasmatic | 14,0 | 17,0 | 8,5 | **0.046** |
|  | membraneous | 11,5 | 2,0 | 6,0 | 0.057 |
| **CDH17** | cytoplasmatic | 4,5 | 13,0 | 6,0 | 0.129 |
|  | membraneous | 11,0 | 14,0 | 4,5 | 0.156 |
| **CDX1** | cytoplasmatic | 0,0 | 0,0 | 0,0 | 0.332 |
|  | nuclear | 0,5 | 1,5 | 0,0 | 0.176 |
| **IP10** | cytoplasmatic | 3,5 | 3,5 | 4,0 | 0.765 |
| **IDO1** | cytoplasmatic | 6,0 | 8,0 | 1,5 | 0.364 |
| **SULF1** | cytoplasmatic | 7,5 | 6,0 | 0,0 | **0.041** |

*Immune-reactivity score (IRS) modified from the method reported by Remmele and Stegner (28). The score represents the product of the intensity of the staining (0=absent, 1=mild, 2=marked, 3=strong) and the proportion of stained cells (0=0%, 10=100%). This was applied to either the staining signal in the cytoplasm, the nucleus or the cell membrane.
