# Supplementary material for: Transcriptomic profiling reveals three molecular phenotypes of adenocarcinoma at the gastroesophageal junction
Source: Int J Cancer. 2019 May 17;145(12):3389–401. doi: 10.1002/ijc.32384 (PMC6851674; doi:10.1002/ijc.32384)
Supplement: Supplementary file 14 — Table S7 Demographic and clinicopathological data of the study cohort (n = 107) [file IJC-145-3389-s014.docx]

**Table 1: Clinico-pathological and epidemiological characteristics of the study cohort**

| Item |  | All (N=107) | **Group 1 (n=28)** | **Group 2 (n=39)** | **Group 3 (n=40)** | p-value |
| --- | --- | --- | --- | --- | --- | --- |
| Age | Median (range), Years | 71.4 (43.9-87.7) | 72.9 (43.9-85.2) | 71.0 (49.74-87.7) | 71.4 (47.5-86.0) | 0.939 |
| Sex | Male (%) | 84 (78.5%) | 25 (89.3%) | 27 (69.2%) | 32 (80.0%) | 0.148 |
| Tumor location | GEJ (%) | 84 (78.5%) | 23 (82.1%) | 33 (84.6%) | 28 (70.0%) | 0.435 |
|  | Body (%) | 15 (14.0%) | 3 (10.7%) | 3 (7.7%) | 9 (32.1%) |  |
|  | Antrum (%) | 8 (7.5%) | 2 (7.1%) | 3 (7.7%) | 3 (7.5%) |  |
| Siewert type (for junctional cancers only) | Type 1 (%) | 35 (41.7%) | 11 (47.8%) | 15 (45.5%) | 9 (32.1%) | 0.107 |
|  | Type 2 (%) | 31 (36.9%) | 11 (47.8%) | 9 (27.3%) | 11 (39.3%) |  |
|  | Type 3 (%) | 18 (16.8%) | 1 (4.3%) | 9 (27.3%) | 8 (28.6%) |  |
| Degree of differentiation | well (%) | 5 (4.7%) | 2 (7.1%) | 2 (5.1%) | 1 (2.5%) | 0.451 |
|  | moderate (%) | 62 (57.9%) | 13 (46.4%) | 26 (66.7%) | 23 (57.5%) |  |
|  | poor (%) | 40 (37.4%) | 13 (46.4%) | 11 (28.2%) | 16 (40.0%) |  |
| UICC Stage^$^ | Stage 1 (%) | 22 (20.6%) | 9 (32.1%) | 8 (20.5%) | 5 (12.5%) | 0.058 |
|  | Stage 2 (%) | 21 (19.6%) | 2 (7.1%) | 12 (30.8%) | 7 (17.5%) |  |
|  | Stage 3 (%) | 47 (43.9%) | 10 (35.7%) | 14 (35.9%) | 23 (57.5%) |  |
|  | Stage 4 (%) | 17 (15.9%) | 7 (25.0%) | 5 (12.8%) | 5 (12.5%) |  |
| T-stage^$^ | T1 (%) | 16 (15.5%) | 7 (25.0%) | 6 (15.4%) | 3 (7.5%) | 0.178 |
|  | T2 (%) | 22 (21.4%) | 4 (14.3%) | 9 (23.1%) | 9 (22.5%) |  |
|  | T3 (%) | 55 (53.4%) | 14 (50.0%) | 22 (56.4%) | 19 (47.5%) |  |
|  | T4 (%) | 10 (9.7%) | 2 (7.1%) | 1 (2.6%) | 7 (17.5%) |  |
| Nodal involvement^$^ | Positive (%) | 69 (67.0%) | 19 (27.5%) | 24 (34.8%) | 26 (37.7%) | 0.865 |
| Distant metastases | Positive (%) | 16 (18.8%) | 7 (43.8%) | 5 (31.3%) | 4 (25.0%) | 0.234 |
| Recurrence after curative resection | (%) | 31 (39.7%) | 10 (32.3%) | 7 (22.6%) | 14 (45.2%) | 0.059 |
| Death at time of the study* | (%) | 60 (56.6%) | 21 (35.0%) | 22 (36.8%) | (17 (28.3%) | **0.036** |
| Curative treatment pathway | (%) | 81 (75.7%) | 19 (23.5%) | 30 (37.0%) | 32 (39.5%) | 0.513 |

# Data on Barrett’s esophagus was not available for the whole study cohort. * *p*-value <0.05 was considered as significant. Three-way ANOVA test was applied for comparison of age, Fisher’s exact test for comparison of categorical variables. $ Tumor stage according to the 7^th^ edition of the UICC TNM classification.
